# Quantitative evidence synthesis: a practical guide on meta-analysis, meta-regression, and publication bias tests for environmental sciences

**DOI:** 10.1186/s13750-023-00301-6

**Published:** 2023-04-24

**Authors:** Shinichi Nakagawa, Yefeng Yang, Erin L. Macartney, Rebecca Spake, Malgorzata Lagisz

**Affiliations:** 1 Evolution & Ecology Research Centre and School of Biological, Earth and Environmental Sciences, University of New South Wales, Sydney, NSW 2052 Australia; 2 Theoretical Sciences Visiting Program, Okinawa Institute of Science and Technology Graduate University, Onna, 904-0495 Japan; 3 School of Biological Sciences, Whiteknights Campus, University of Reading, Reading, RG6 6AS UK

**Keywords:** Hierarchical models, Robust variance estimation, Spatial dependency, Variance–covariance matrix, Meta-analysis of variance, Missing data, Network meta-analysis, Multivariate meta-analysis

## Abstract

Meta-analysis is a quantitative way of synthesizing results from multiple studies to obtain reliable evidence of an intervention or phenomenon. Indeed, an increasing number of meta-analyses are conducted in environmental sciences, and resulting meta-analytic evidence is often used in environmental policies and decision-making. We conducted a survey of recent meta-analyses in environmental sciences and found poor standards of current meta-analytic practice and reporting. For example, only ~ 40% of the 73 reviewed meta-analyses reported heterogeneity (variation among effect sizes beyond sampling error), and publication bias was assessed in fewer than half. Furthermore, although almost all the meta-analyses had multiple effect sizes originating from the same studies, non-independence among effect sizes was considered in only half of the meta-analyses. To improve the implementation of meta-analysis in environmental sciences, we here outline practical guidance for conducting a meta-analysis in environmental sciences. We describe the key concepts of effect size and meta-analysis and detail procedures for fitting multilevel meta-analysis and meta-regression models and performing associated publication bias tests. We demonstrate a clear need for environmental scientists to embrace multilevel meta-analytic models, which explicitly model dependence among effect sizes, rather than the commonly used random-effects models. Further, we discuss how reporting and visual presentations of meta-analytic results can be much improved by following reporting guidelines such as PRISMA-EcoEvo (Preferred Reporting Items for Systematic Reviews and Meta-Analyses for Ecology and Evolutionary Biology). This paper, along with the accompanying online tutorial, serves as a practical guide on conducting a complete set of meta-analytic procedures (i.e., meta-analysis, heterogeneity quantification, meta-regression, publication bias tests and sensitivity analysis) and also as a gateway to more advanced, yet appropriate, methods.

**Supplementary Information:**

The online version contains supplementary material available at 10.1186/s13750-023-00301-6.

## Background

Evidence synthesis is an essential part of science. The method of systematic review provides the most trusted and unbiased way to achieve the synthesis of evidence [1–3]. Systematic reviews often include a quantitative summary of studies on the topic of interest, referred to as a meta-analysis (for discussion on the definitions of ‘meta-analysis’, see [4]). The term meta-analysis can also mean a set of statistical techniques for quantitative data synthesis. The methodologies of the meta-analysis were initially developed and applied in medical and social sciences. However, meta-analytic methods are now used in many other fields, including environmental sciences [5–7]. In environmental sciences, the outcomes of meta-analyses (within systematic reviews) have been used to inform environmental and related policies (see [8]). Therefore, the reliability of meta-analytic results in environmental sciences is important beyond mere academic interests; indeed, incorrect results could lead to ineffective or sometimes harmful environmental policies [8].

As in medical and social sciences, environmental scientists frequently use traditional meta-analytic models, namely fixed-effect and random-effects models [9, 10]. However, we contend that such models in their original formulation are no longer useful and are often incorrectly used, leading to unreliable estimates and errors. This is mainly because the traditional models assume independence among effect sizes, but almost all primary research papers include more than one effect size, and this non-independence is often not considered (e.g., [11–13]). Furthermore, previous reviews of published meta-analyses in environmental sciences (hereafter, ‘environmental meta-analyses’) have demonstrated that less than half report or investigate heterogeneity (inconsistency) among effect sizes [14–16]. Many environmental meta-analyses also do not present any sensitivity analysis, for example, for publication bias (i.e., statistically significant effects being more likely to be published, making collated data unreliable; [17, 18]). These issues might have arisen for several reasons, for example, because of no clear conduct guideline for the statistical part of meta-analyses in environmental sciences and rapid developments in meta-analytic methods. Taken together, the field urgently requires a practical guide to implement correct meta-analyses and associated procedures (e.g., heterogeneity analysis, meta-regression, and publication bias tests; cf. [19]).

To assist environmental scientists in conducting meta-analyses, the aims of this paper are five-fold. First, we provide an overview of the processes involved in a meta-analysis while introducing some key concepts. Second, after introducing the main types of effect size measures, we mathematically describe the two commonly used traditional meta-analytic models, demonstrate their utility, and introduce a practical, multilevel meta-analytic model for environmental sciences that appropriately handles non-independence among effect sizes. Third, we show how to quantify heterogeneity (i.e., consistencies among effect sizes and/or studies) using this model, and then explain such heterogeneity using meta-regression. Fourth, we show how to test for publication bias in a meta-analysis and describe other common types of sensitivity analysis. Fifth, we cover other technical issues relevant to environmental sciences (e.g., scale and phylogenetic dependence) as well as some advanced meta-analytic techniques. In addition, these five aims (sections) are interspersed with two more sections, named ‘Notes’ on: (1) visualisation and interpretation; and (2) reporting and archiving. Some of these sections are accompanied by results from a survey of 73 environmental meta-analyses published between 2019 and 2021; survey results depict current practices and highlight associated problems (for the method of the survey, see Additional file 1). Importantly, we provide easy-to-follow implementations of much of what is described below, using the *R* package, *metafor* [20] and other *R* packages at the webpage (https://itchyshin.github.io/Meta-analysis_tutorial/), which also connects the reader to the wealth of online information on meta-analysis (note that we also provide this tutorial as Additional file 2; see also [21]).

## Overview with key concepts

Statistically speaking, we have three general objectives when conducting a *meta-analysis* [12]: (1) estimating *an overall mean*, (2) quantifying consistency (*heterogeneity*) between studies, and (3) explaining the heterogeneity (see Table 1 for the definitions of the terms in *italic*). A notable feature of a meta-analysis is that an overall mean is estimated by taking the *sampling variance* of each *effect size* into account: a study (effect size) with a low sampling variance (usually based on a larger sample size) is assigned more weight in estimating an overall mean than one with a high sampling variance (usually based on a smaller sample size). However, an overall mean estimate itself is often not informative because one can get the same overall mean estimates in different ways. For example, we may get an overall estimate of zero if all studies have zero effects with no heterogeneity. In contrast, we might also obtain a zero mean across studies that have highly variable effects (e.g., ranging from strongly positive to strongly negative), signifying high heterogeneity. Therefore, quantifying indicators of heterogeneity is an essential part of a meta-analysis, necessary for interpreting the overall mean appropriately. Once we observe non-zero heterogeneity among effect sizes, then, our job is to explain this variation by running *meta-regression* models, and, at the same time, quantify how much variation is accounted for (often quantified as *R*^2^). In addition, it is important to conduct an extra set of analyses, often referred to as *publication bias tests*, which are a type of *sensitivity analysis* [11], to check the robustness of meta-analytic results.

**Table 1:** Definitions of key concepts and associated statistical parameters, which are used in formulas in the main text

Term	Definition (with associated parameters, if any)
Effect size	A measurement of effect (usually state of a single group, comparison between groups, or association, see Table 2). In a meta-analytic model, it becomes the response variable (noted as *z*_*i*_ in the formulas)
Sampling variance	A measure of uncertainty in effect size (noted as *v*_*i*_). Its inverse is often called ‘weight’ (the square-root of weight is ‘precision’, and the square root of sampling variance is ‘sampling standard error’)
Meta-analysis	A statistical method to aggregate effect sizes from studies on the same or similar topics, by assigning different weights based on sampling variance of effect sizes. Strictly speaking, in a formal (weighted) meta-analysis, sampling variance needs to be incorporated and it is assumed to be known (Table 2)
Overall mean (effect)	An average effect size based on a meta-analytic model (noted as ${\beta }_{0}$ and its standard errors, se(${\beta }_{0}$))
Heterogeneity	An indicator of consistency among effect sizes, or an extent of variation around the overall effect (${\beta }_{0}$); heterogeneity can be quantified by absolute measures, such as ${\tau }^{2}$, or relative measures, such as *I*^2^
Meta-regression	A regression model which extends a meta-analytic model with a moderator(s), aiming to explain heterogeneity (quantified as *R*^2^) and quantifying the effect of a moderator (noted as, for example, ${\beta }_{1}$)
Publication bias tests	A set of statistical methodologies to detect and correct for publication bias, where a subset of results (positive findings) is more likely to be published and present in the meta-analytic dataset than otherwise
Sensitivity analysis	A set of statistical analyses that checks the robustness of one’s main analysis; if sensitivity analysis shows different results (qualitatively and/or quantitively), then we must doubt the robustness of the main findings

## Choosing an effect size measure

In this section, we introduce different kinds of ‘effect size measures’ or ‘effect measures’. In the literature, the term ‘effect size’ is typically used to refer to the magnitude or strength of an effect of interest or its biological interpretation (e.g., environmental significance). Effect sizes can be quantified using a range of measures (for details, see [22]). In our survey of environmental meta-analyses (Additional file 1), the two most commonly used effect size measures are: the logarithm of response ratio, lnRR ([23]; also known as the ratio of means; [24]) and standardized mean difference, SMD (often referred to as Hedges’ *g* or Cohen’s *d* [25, 26]). These are followed by proportion (%) and Fisher’s *z*-transformation of correlation, or *Zr*. These four effect measures nearly fit into the three categories, which are named: (1) single-group measures (a statistical summary from one group; e.g., proportion), (2) comparative measures (comparing between two groups e.g., SMD and lnRR), and (3) association measures (relationships between two variables; e.g., *Zr*). Table 2 summarizes effect measures that are common or potentially useful for environmental scientists. It is important to note that any measures with sampling variance can become an ‘effect size’. The main reason why SMD, lnRR, *Zr,* or proportion are popular effect measures is that they are unitless, while a meta-analysis of mean, or mean difference, can only be conducted when all effect sizes have the same unit (e.g., cm, kg).

**Table 2:** Selected list of effect size measures and their sampling variances, belonging to three types: (1) single-group effect, (2) comparative effect and (3) association effect

Type	Effect size	Point estimate	Sampling variance estimate	Reference
Single group	Mean	$\overline{x}_{i}$	${s}_{i}^{2}/{n}_{i}$	[134]
Single group	Proportion	${p}_{i}=\frac{{y}_{i}}{{n}_{i}}$	$\frac{{p}_{i}\left(1-{p}_{i}\right)}{{n}_{i}}=\frac{{y}_{i}\left({n}_{i}-{y}_{i}\right)}{{n}_{i}^{3}}$	[134]
Single group	Log standard deviation (lnSD)	${\text{ln}}{s}_{i}$	$\frac{1}{2\left({n}_{i}-1\right)}$	[27]
Single group	Log coefficient of variation (lnCV)	$\ln \left( {\frac{{S_{i} }}{{\overline{x}_{i} }}} \right)$	$\frac{{s_{i}^{2} }}{{n_{i} \overline{x}_{i}^{2} }} + \frac{1}{{2\left( {n_{i} - 1} \right)}}$	[27]
Comparative	Mean difference (MD)	$\overline{x}_{iT} - \overline{x}_{iC}$	$\frac{{s}_{iC}^{2}}{{n}_{iC}}+\frac{{s}_{iT}^{2}}{{n}_{iT}}$	[134]
Comparative	Standardised mean difference (SMD)	$d_{i} = \frac{{\overline{x}_{iT} - \overline{x}_{iC} }}{{\sqrt {\frac{{\left( {n_{iC} - 1} \right)s_{iC}^{2} + \left( {n_{iT} - 1} \right)s_{iT}^{2} }}{{n_{iC} + n_{iT} - 2}}} }}$	$\frac{1}{{n}_{iC}}+\frac{1}{{n}_{iT}}+\frac{{d}_{i}^{2}}{2\left({n}_{iC}+{n}_{iT}\right)}$	[25]
Comparative	Risk (proportion) difference (RD)	$\frac{{y}_{iT}}{{n}_{iT}}-\frac{{y}_{iC}}{{n}_{iC}}$	$\frac{{y}_{iT}\left({n}_{iT}-{y}_{iT}\right)}{{n}_{iT}^{3}}+\frac{{y}_{iC}\left({n}_{iC}-{y}_{iC}\right)}{{n}_{iC}^{3}}$	[134]
Comparative	Log odds ratio (lnOR)	${\text{ln}}\left(\frac{{y}_{iT}}{{n}_{iT}-{y}_{iT}}\right)-{\text{ln}}\left(\frac{{y}_{iC}}{{n}_{iC}-{y}_{iC}}\right)$	$\frac{1}{{y}_{iT}}+\frac{1}{{n}_{iT}-{y}_{iT}}+\frac{1}{{y}_{iC}}+\frac{1}{{n}_{iC}-{y}_{iC}}$	[134]
Comparative	Log response ratio (lnRR)	${\text{ln}}\left( {\frac{{\overline{x}_{iT} }}{{\overline{x}_{iC} }}} \right)$	$\frac{{s_{iC}^{2} }}{{n_{iC} \overline{x}_{iC}^{2} }} + \frac{{s_{iT}^{2} }}{{n_{iT} \overline{x}_{iT}^{2} }}$	[135]
Comparative	Log variability ratio (lnVR)	${\text{ln}}\left(\frac{{s}_{iT}}{{s}_{iC}}\right)$	$\frac{1}{2\left({n}_{iC}-1\right)}+\frac{1}{2\left({n}_{iT}-1\right)}$	[27]
Comparative	Log coefficient of variation ratio (lnCVR)	${\text{ln}}\left( {\frac{{s_{iT} }}{{\overline{x}_{iT} }}} \right) - {\text{ln}}\left( {\frac{{s_{iC} }}{{\overline{x}_{iC} }}} \right)$	$\frac{{s_{iC}^{2} }}{{n_{iC} \overline{x}_{iC}^{2} }} + \frac{1}{{2\left( {n_{iC} - 1} \right)}} + \frac{{s_{iT}^{2} }}{{n_{iT} \overline{x}_{iT}^{2} }} + \frac{1}{{2\left( {n_{iT} - 1} \right)}}$	[27]
Association	Fisher’s z-transformation of correlation, *r* (*Zr*)	$\frac{1}{2}{\text{ln}}\left(\frac{1+{r}_{i}}{1-{r}_{i}}\right)$	$\frac{1}{{n}_{i}-3}$	[134]

For the column 3rd and 4th, notations represent: $\overline{x}$ (mean), *s* (standard deviation), *n* (sampling size), *y* (the number of events), the subscript *T* (treatment group), the subscript *C* (control group) and the subscript *i* (the *i*th effect size or study)

Note that better estimators may be found in the relevant references; for example, SMD can be best estimated by multiplying by $\left(1-\frac{3}{4\left({n}_{iC}+{n}_{iT}-2\right)-1}\right)$, and see also [43]

Table 2 also includes effect measures that are likely to be unfamiliar to environmental scientists; these are effect sizes that characterise differences in the observed variability between samples, (i.e., lnSD, lnCV, lnVR and lnCVR; [27, 28]) rather than central tendencies (averages). These dispersion-based effect measures can provide us with extra insights along with average-based effect measures. Although the literature survey showed none of these were used in our sample, these effect sizes have been used in many fields, including agriculture (e.g., [29]), ecology (e.g., [30]), evolutionary biology (e.g., [31]), psychology (e.g., [32]), education (e.g., [33]), psychiatry (e.g., [34]), and neurosciences (e.g. [35],),. Perhaps, it is not difficult to think of an environmental intervention that can affect not only the mean but also the variance of measurements taken on a group of individuals or a set of plots. For example, environmental stressors such as pesticides and eutrophication are likely to increase variability in biological systems because stress accentuates individual differences in environmental responses (e.g. [36, 37],). Such ideas are yet to be tested meta-analytically (cf. [38, 39]).

## Choosing a meta-analytic model

### Fixed-effect and random-effects models

**Fig. 1:**
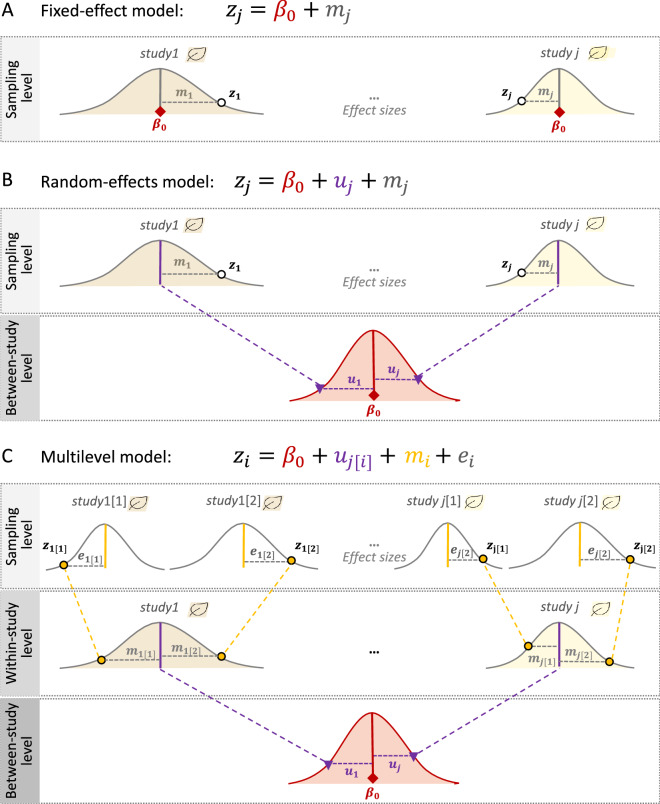
Visualisation of the three statistical models of meta-analysis: **A** a fixed-effect model (1-level), **B** a random-effects model (2-level), and **C** a multilevel model (3-level; see the text for what symbols mean)

Two traditional meta-analytic models are called the ‘fixed-effect’ model and the ‘random-effects’ model. The former assumes that all effect sizes (from different studies) come from one population (i.e., they have one true overall mean), while the latter does not have such an assumption (i.e., each study has different overall means or heterogeneity exists among studies; see below for more). The fixed-effect model, which should probably be more correctly referred to as the ‘common-effect’ model, can be written as [9, 10, 40]:

1 $${z}_{j}={\beta }_{0}+{m}_{j},$$

$${m}_{j}\sim \mathrm{N}\left(0,{v}_{j}\right),$$

where the intercept, ${\beta }_{0}$ is the overall mean, *z*_*j*_ (the response/dependent variable) is the effect size from the *j*th study (*j* = 1, 2,…, *N*_*study*_; in this model, *N*_*study*_ = the number of studies = the number of effect sizes), *m*_*j*_ is the sampling error, related to the *j*th sampling variance (*v*_*j*_), which is normally distributed with the mean of 0 and the ‘study-specific’ sampling variance, *v*_*j*_ (see also Fig. 1A).

The overall mean needs to be estimated and often done so as the weighted average with the weights, ${w}_{j}=1/{v}_{j}$ (i.e., the inverse-variance approach). An important, but sometimes untenable, assumption of meta-analysis is that sampling variance is known. Indeed, we estimate sampling variance, using formulas, as in Table 2, meaning that vj is submitted by sampling variance estimates (see also section ‘Scale dependence’). Of relevance, the use of the inverse-variance approach has been recently criticized, especially for SMD and lnRR [41, 42] and we note that the inverse-variance approach using the formulas in Table 2 is one of several different weighting approaches used in meta-analysis (e.g., for adjusted sampling-variance weighing, see [43, 44]; for sample-size-based weighting, see [41, 42, 45, 46]). Importantly, the fixed-effect model assumes that the only source of variation in effect sizes (*z*_*j*_) is the effect due to sampling variance (which is inversely proportional to the sample size, *n*; Table 2).

Similarly, the random-effects model can be expressed as:

2 $${z}_{j}={\beta }_{0}+{u}_{j}+{m}_{j},$$

$${u}_{j}\sim \mathrm{N}\left(0,{\tau }^{2}\right), \, \& \  {m}_{j}\sim \mathrm{N}\left(0,{v}_{j}\right),$$

where *u*_*j*_ is the *j*th study effect, which is normally distributed with the mean of 0 and the between-study variance, ${\tau }^{2}$ (for different estimation methods, see [47–50]), and other notations are the same as in Eq. 1 (Fig. 1B). Here, the overall mean can be estimated as the weighted average with weights ${w}_{j}=1/\left({\tau }^{2}+{v}_{j}^{2}\right)$ (note that different weighting approaches, mentioned above, are applicable to the random-effects model and some of them are to the multilevel model, introduced below). The model assumes each study has its specific mean, ${b}_{0}+{u}_{j}$, and (in)consistencies among studies (effect sizes) are indicated by ${\tau }^{2}$. When ${\tau }^{2}$ is 0 (or not statistically different from 0), the random-effects model simplifies to the fixed-effect model (cf. Equations 1 and 2). Given no studies in environmental sciences are conducted in the same manner or even at exactly the same place and time, we should expect different studies to have different means. Therefore, in almost all cases in the environmental sciences, the random-effects model is a more ‘realistic’ model [9, 10, 40]. Accordingly, most environmental meta-analyses (68.5%; 50 out of 73 studies) in our survey used the random-effects model, while only 2.7% (2 of 73 studies) used the fixed-effect model (Additional file 1).

### Multilevel meta-analytic models

Although we have introduced the random-effects model as being more realistic than the fixed-effect model (Eq. 2), we argue that the random-effects model is rather limited and impractical for the environmental sciences. This is because random-effects models, like fixed-effect models, assume all effect sizes (*z*_*j*_) to be independent. However, when multiple effect sizes are obtained from a study, these effect sizes are dependent (for more details, see the next section on non-independence). Indeed, our survey showed that in almost all datasets used in environmental meta-analyses, this type of non-independence among effect sizes occurred (97.3%; 71 out of 73 studies, with two studies being unclear, so effectively 100%; Additional file 1). Therefore, we propose the simplest and most practical meta-analytic model for environmental sciences as [13, 40] (see also [51, 52]):

3 $${z}_{i}={\beta }_{0}+{u}_{j\left[i\right]}+{e}_{i}+{m}_{i},$$

$${u}_{j}\sim \mathrm{N}\left(0,{\tau }^{2}\right), { e}_{i}\sim \mathrm{N}\left(0,{\sigma }^{2}\right), \, \& \ {m}_{i}\sim \mathrm{N}\left(0,{v}_{i}\right)$$

where we explicitly recognize that *N*_*effect*_ (*i* = 1, 2,…, *N*_*effect*_) > *N*_*study*_ (*j* = 1, 2,…, *N*_*study*_) and, therefore, we now have the study effect (between-study effect), *u*_j[i]_ (for the *j*th study and *i*th effect size) and effect-size level (within-study) effect, *e*_*i*_ (for the *i*th effect size), with the between-study variance, ${\tau }^{2}$, and with-study variance, ${\sigma }^{2}$, respectively, and other notations are the same as above. We note that this model (Eq. 3) is an extension of the random-effects model (Eq. 2), and we refer to it as the multilevel/hierarchical model (used in 7 out of 73 studies: 9.6% [Additional file 1]; note that Eq. 3 is also known as a three-level meta-analytic model; Fig. 1C). Also, environmental scientists who are familiar with (generalised) linear mixed-models may recognize *u*_*j*_ (the study effect) as the effect of a random factor which is associated with a variance component, i.e., ${\tau }^{2}$ [53]; also, *e*_*i*_ and *m*_*i*_ can be seen as parts of random factors, associated with ${\sigma }^{2}$ and *v*_*i*_ (the former is comparable to the residuals, while the latter is sampling variance, specific to a given effect size).

It seems that many researchers are aware of the issue of non-independence so that they often use average effect sizes per study or choose one effect size (at least 28.8%, 21 out of 73 environmental meta-analyses; Additional file 1). However, as we discussed elsewhere [13, 40], such averaging or selection of one effect size per study dramatically reduces our ability to investigate environmental drivers of variation among effect sizes [13]. Therefore, we strongly support the use of the multilevel model. Nevertheless, this proposed multilevel model, formulated as Eq. 3 does not usually deal with the issue of non-independence completely, which we elaborate on in the next section.

### Non-independence among effect sizes and among sampling errors

**Fig. 2:**
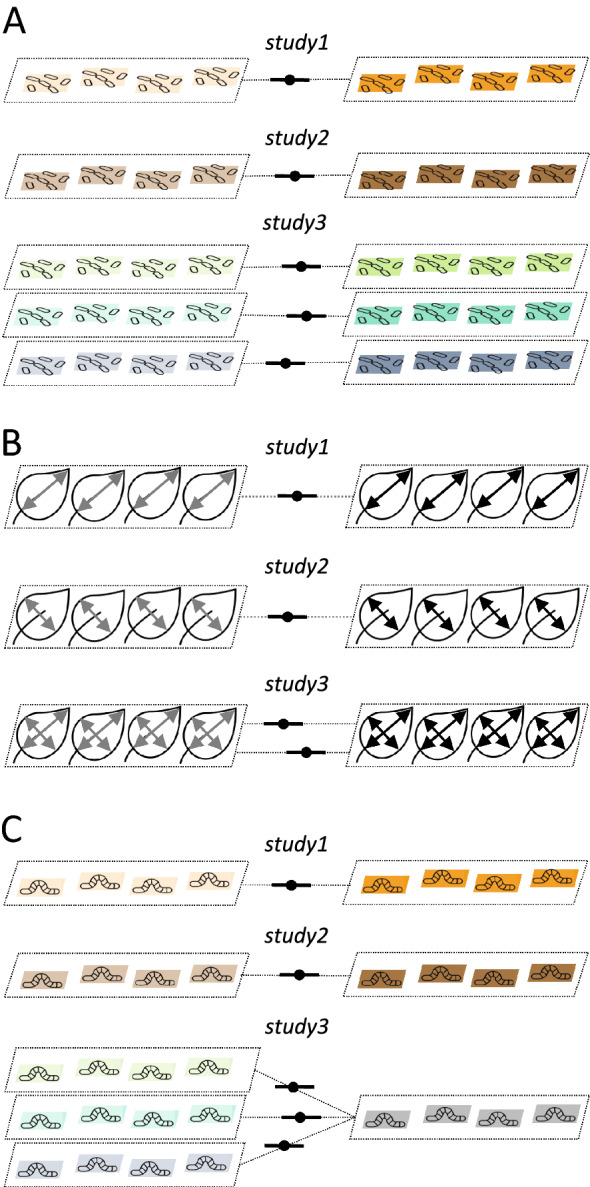
Visualisation of the three types of non-independence among effect sizes: **A** due to shared study identities (effect sizes from the same study), **B** due to shared measurements (effect sizes come from the same group of individuals/plots but are based on different types of measurements), and **C** due to shared control (effect sizes are calculated using the same control group and multiple treatment groups; see the text for more details)

When you have multiple effect sizes from a study, there are two broad types and three cases of non-independence (cf. [11, 12]): (1) effect sizes are calculated from different cohorts of individuals (or groups of plots) within a study (Fig. 2A, referred to as ‘shared study identity’), and (2) effects sizes are calculated from the same cohort of individuals (or group of plots; Fig. 2B, referred to as ‘shared measurements’) or *partially* from the same individuals and plots, more concretely, sharing individuals and plots from the control group (Fig. 2C, referred to as ‘shared control group’). The first type of non-independence induces dependence among effect sizes, but not among sampling variances, and the second type leads to non-independence among sampling variances. Many datasets, if not almost all, will have a combination of these three cases (or even are more complex, see the section "Complex non-independence"). Failing to deal with these non-independences will inflate Type 1 error (note that the overall estimate, *b*_0_ is unlikely to be biased, but standard error of *b*_0_, se(*b*_0_), will be underestimated; note that this is also true for all other regression coefficients, e.g., *b*_1_; see Table 1). The multilevel model (as in Eq. 3) only takes care of cases of non-independence that are due to the shared study identity but neither shared measurements nor shared control group.

There are two practical ways to deal with non-independence among sampling variances. The first method is that we explicitly model such dependence using a variance–covariance (VCV) matrix (used in 6 out of 73 studies: 8.2%; Additional file 1). Imagine a simple scenario with a dataset of three effect sizes from two studies where two effects sizes from the first study are calculated (partially) using the same cohort of individuals (Fig. 2B); in such a case, the sampling variance effect, ${m}_{i}$, as in Eq. 3, should be written as:

$${m}_{i}\sim \mathrm{N}\left(0,\mathbf{M}\right)$$

4 $$\mathbf{M}=\left[\begin{array}{ccc}{v}_{1\left[1\right]}& \rho \sqrt{{v}_{1\left[1\right]}{v}_{1\left[2\right]}}& 0\\ \rho \sqrt{{v}_{1\left[2\right]}{v}_{1\left[1\right]}}& {v}_{1\left[2\right]}& 0\\ 0& 0& {v}_{2\left[3\right]}\end{array}\right],$$

where **M** is the VCV matrix showing the sampling variances,${v}_{1\left[1\right]}$ (study 1 and effect size 1), ${v}_{1\left[2\right]}$ (study 1 and effect size 2), and ${v}_{2\left[3\right]}$ (study 2 and effect size 3) in its diagonal, and sampling covariance, $\rho \sqrt{{v}_{1\left[1\right]}{v}_{1\left[2\right]}}= \rho \sqrt{{v}_{1\left[2\right]}{v}_{1\left[1\right]}}$ in its off-diagonal elements, where $\rho$ is a correlation between two sampling variances due to shared samples (individuals/plots). Once this VCV matrix is incorporated into the multilevel model (Eq. 3), all the types of non-independence, as in Fig. 2, are taken care of. Table 3 shows formulas for the sampling variance and covariance of the four common effect sizes (SDM, lnRR, proportion and *Zr*). For comparative effect measures (Table 2), exact covariances can be calculated under the case of ‘shared control group’ (see [54, 55]). But this is not feasible for most circumstances because we usually do not know what $\rho$ should be. Some have suggested fixing this value at 0.5 (e.g., [11]) or 0.8 (e.g., [56]); the latter is a more conservative assumption. Or one can run both and use one for the main analysis and the other for sensitivity analysis (for more, see the ‘Conducting sensitivity analysis and critical appraisal" section).

**Table 3:** Examples of dependence between two sampling variances (*v*_1_ and *v*_2_) and their covariance for four common effect size statistics

Effect size	Situation	Variances estimate	Covariance estimate
Proportion	Shared measurement	${v}_{1}=\frac{{y}_{1}\left({n}_{1}-{y}_{1}\right)}{{n}_{1}^{3}}$ ${v}_{2}=\frac{{y}_{2}\left({n}_{2}-{y}_{2}\right)}{{n}_{2}^{3}}$	$\rho \sqrt{\frac{{y}_{1}\left({n}_{1}-{y}_{1}\right)}{{n}_{1}^{3}}\frac{{y}_{2}\left({n}_{2}-{y}_{2}\right)}{{n}_{2}^{3}}}$
*Zr*	Shared measurement	${v}_{1}=\frac{1}{2}{\text{ln}}\left(\frac{1+{r}_{1}}{1-{r}_{1}}\right)$ ${v}_{2}=\frac{1}{2}{\text{ln}}\left(\frac{1+{r}_{2}}{1-{r}_{2}}\right)$	$\rho \sqrt{\frac{1}{4}{\text{ln}}\left(\frac{1+{r}_{1}}{1-{r}_{1}}\right){\text{ln}}\left(\frac{1+{r}_{2}}{1-{r}_{2}}\right)}$
lnRR	Shared measurement	$v_{1} = \frac{{s_{1C}^{2} }}{{n_{1C} \overline{x}_{1C}^{2} }} + \frac{{s_{1T}^{2} }}{{n_{1T} \overline{x}_{1T}^{2} }}$ $v_{2} = \frac{{s_{2C}^{2} }}{{n_{2C} \cdot \overline{x}_{2C}^{2} }} + \frac{{s_{2T}^{2} }}{{n_{2T} \cdot \overline{x}_{2T}^{2} }}$	$\rho \sqrt {\left( {\frac{{s_{1C}^{2} }}{{n_{1C} \overline{x}_{1C}^{2} }} + \frac{{s_{1T}^{2} }}{{n_{1T} \overline{x}_{1T}^{2} }}} \right)\left( {\frac{{s_{2C}^{2} }}{{n_{2C} \overline{x}_{2C}^{2} }} + \frac{{s_{2T}^{2} }}{{n_{2T} \overline{x}_{2T}^{2} }}} \right)}$
	Shared control	$v_{1} = \frac{{s_{1C}^{2} }}{{n_{1C} \overline{x}_{1C}^{2} }} + \frac{{s_{1T}^{2} }}{{n_{1T} \overline{x}_{1T}^{2} }}$ $v_{2} = \frac{{s_{1C}^{2} }}{{n_{1C} \overline{x}_{1C}^{2} }} + \frac{{s_{2T}^{2} }}{{n_{2T} \overline{x}_{2T}^{2} }}$	$\frac{{s_{1C}^{2} }}{{n_{1C} \overline{x}_{1C}^{2} }}$
SMD	Shared measurement	${v}_{1}=\frac{1}{{n}_{1C}}+\frac{1}{{n}_{1T}}+\frac{{d}_{1}^{2}}{2\left({n}_{1C}+{n}_{1T}\right)}$ ${v}_{2}=\frac{1}{{n}_{2C}}+\frac{1}{{n}_{2T}}+\frac{{d}_{1}^{2}}{2\left({n}_{2C}+{n}_{2T}\right)}$	$\rho \sqrt{\left(\frac{1}{{n}_{1C}}+\frac{1}{{n}_{1T}}+\frac{{d}_{1}^{2}}{2\left({n}_{1C}+{n}_{1T}\right)}\right)\left(\frac{1}{{n}_{2C}}+\frac{1}{{n}_{2T}}+\frac{{d}_{2}^{2}}{2\left({n}_{2C}+{n}_{2T}\right)}\right)}$
	Shared control	${v}_{1}=\frac{1}{{n}_{1C}}+\frac{1}{{n}_{1T}}+\frac{{d}_{1}^{2}}{2\left({n}_{1C}+{n}_{1T}+{n}_{2T}\right)}$ ${v}_{2}=\frac{1}{{n}_{1C}}+\frac{1}{{n}_{2T}}+\frac{{d}_{2}^{2}}{2\left({n}_{1C}+{n}_{1T}+{n}_{2T}\right)}$	$\frac{1}{{n}_{1C}}+\frac{{d}_{1}{d}_{2}}{2\left({n}_{1C}+{n}_{1T}+{n}_{2T}\right)}$

For the 2nd column, see Fig. 2. For the 3rd and 4th column, notations represent: the subscript 1*C* and 2*C* (control group for 1st and 2nd effect size, respectively, but for shared control, 1*C* is used for both effect sizes, but 1*C* and 2*C* are the same cohort or set of plots), the subscript *1T* and 2*T* (treatment group for the 1st and 2nd effect size, respectively; for shared groups, 1T and 2T represents different groups of individuals/plots whereas, for shared measurements, 1T and 2T are the same set of individuals/plots), $\rho$ is a correlation in sampling error variance between two measurements, and the other notations are as in Table 1 and the main text (see also [54, 55])

The second method overcomes this very issue of unknown $\rho$ by approximating average dependence among sampling variance (and effect sizes) from the data and incorporating such dependence to estimate standard errors (only used in 1 out of 73 studies; Additional file 1). This method is known as ‘robust variance estimation’, RVE, and the original estimator was proposed by Hedges and colleagues in 2010 [57]. Meta-analysis using RVE is relatively new, and this method has been applied to multilevel meta-analytic models only recently [58]. Note that the random-effects model (Eq. 2) and RVE could correctly model both types of non-independence. However, we do not recommend the use of RVE with Eq. 2 because, as we will later show, estimating ${\sigma }^{2}$ as well as ${\tau }^{2}$ will constitute an important part of understanding and gaining more insights from one’s data. We do not yet have a definite recommendation on which method to use to account for non-independence among sampling errors (using the VCV matrix or RVE). This is because no simulation work in the context of multilevel meta-analysis has been done so far, using multilevel meta-analyses [13, 58]. For now, one could use both VCV matrices and RVE in the same model [58] (see also [21]).

## Quantifying and explaining heterogeneity

### Measuring consistencies with heterogeneity

As mentioned earlier, quantifying heterogeneity among effect sizes is an essential component of any meta-analysis. Yet, our survey showed only 28 out of 73 environmental meta-analyses (38.4%; Additional file 1) report at least one index of heterogeneity (e.g., ${\tau }^{2}$, *Q*, and *I*^2^). Conventionally, the presence of heterogeneity is tested by Cochrane’s *Q* test. However, *Q* (often noted as *Q*_*T*_ or *Q*_*total*_), and its associated *p* value, are not particularly informative: the test does not tell us about the extent of heterogeneity (e.g. [10],), only whether heterogeneity is zero or not (when *p* < 0.05). Therefore, for environmental scientists, we recommend two common ways of quantifying heterogeneity from a meta-analytic model: absolute heterogeneity measure (i.e., variance components, ${\tau }^{2}$ and ${\sigma }^{2}$) and relative heterogeneity measure (i.e., *I*^2^; see also the "Notes on visualisation and interpretation" section for another way of quantifying and visualising heterogeneity at the same time, using prediction intervals; see also [59]). We have already covered the absolute measure (Eqs. 2 & 3), so here we explain *I*^2^, which ranges from 0 to 1 (for some caveats for *I*^2^, see [60, 61]). The heterogeneity measure, *I*^2^, for the random-effect model (Eq. 2) can be written as:

5 $$ I^{2} = \frac{{\tau^{2} }}{{\tau^{2} + \overline{v} }}, $$

6 $$ \overline{v} = \frac{{\left( {N_{effect} - 1} \right)\mathop \sum \nolimits_{j = 1}^{k} \left( {1/v_{i} } \right)}}{{\left( {\mathop \sum \nolimits_{j = 1}^{k} \left( {1/v_{i} } \right)} \right)^{2} - \mathop \sum \nolimits_{j = 1}^{k} \left( {1/v_{i} } \right)^{2} }}, $$

Where $\overline{v}$ is referred to as the typical sampling variance (originally this is called ‘within-study’ variance, as in Eq. 2, and note that in this formulation, within-study effect and the effect of sampling error is confounded; see [62, 63]; see also [64]) and the other notations are as above. As you can see from Eq. 5, we can interpret *I*^2^ as relative variation due to differences between studies (between-study variance) or relative variation not due to sampling variance.

By seeing *I*^2^ as a type of interclass correlation (also known as repeatability [65],), we can generalize *I*^2^ to multilevel models. In the case of Eq. 3 ([40, 66]; see also [52]), we have:

7 $$ I_{total}^{2} = \frac{{\tau^{2} + \sigma^{2} }}{{\tau^{2} + \sigma^{2} + \overline{v} }} $$

Because we can have two more *I*^2^, Eq. 7 is written as ${I}_{total}^{2}$; these other two are ${I}_{study}^{2}$ and ${I}_{effect}^{2}$, respectively:

8 $$ I_{study}^{2} = \frac{{\tau^{2} }}{{\tau^{2} + \sigma^{2} + \overline{v} }}, $$

9 $$ I_{effect}^{2} = \frac{{\sigma^{2} }}{{\tau^{2} + \sigma^{2} + \overline{v} }} $$

**Fig. 3:**
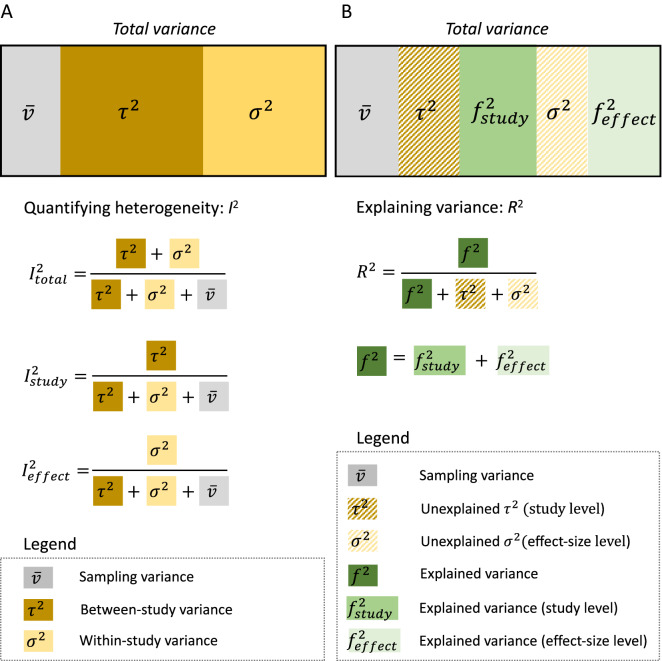
Visualisation of variation (heterogeneity) partitioned into different variance components: **A** quantifying different types of *I*^2^ from a multilevel model (3-level; see Fig. 1C) and **B** variance explained, *R*^2^, by moderators. Note that different levels of variances would be explained, depending on which level a moderator belongs to (study level and effect-size level)

${I}_{total}^{2}$ represents relative variance due to differences both between and within studies (between- and within-study variance) or relative variation not due to sampling variance, while ${I}_{study}^{2}$ is relative variation due to differences between studies, and ${I}_{effect}^{2}$ is relative variation due to differences within studies (Fig. 3A). Once heterogeneity is quantified (note almost all data will have non-zero heterogeneity and an earlier meta-meta-analysis suggests in ecology, we have on average, *I*^2^ close to 90% [66]), it is time to fit a meta-regression model to explain the heterogeneity. Notably, the magnitude of ${I}_{study}^{2}$ (and ${\tau }^{2}$) and ${I}_{effect}^{2}$ (and ${\sigma }^{2}$) can already inform you which predictor variable (usually referred to as ‘moderator’) is likely to be important, which we explain in the next section.

### Explaining variance with meta-regression

We can extend the multilevel model (Eq. 3) to a meta-regression model with one moderator (also known as predictor, independent, explanatory variable, or fixed factor), as below:

10 $${z}_{i}={\beta }_{0}+{\beta }_{1}{x}_{1j\left[i\right]}+{u}_{j\left[i\right]}+{e}_{i}+{m}_{i},$$

where ${\beta }_{1}$ is a slope of the moderator (*x*_1_), ${x}_{1j\left[i\right]}$ denotes the value of *x*_1_, corresponding to the *j*th study (and the *i*th effect sizes). Equation (10) (meta-regression) is comparable to the simplest regression with the intercept (${\beta }_{0}$) and slope (${\beta }_{1}$). Notably, ${x}_{1j\left[i\right]}$ differs between studies and, therefore, it will mainly explain the variance component, ${\tau }^{2}$ (which relates to ${I}_{study}^{2}$). On the other hand, if noted like ${x}_{1i}$, this moderator would vary within studies or at the level of effect sizes, therefore, explaining ${\sigma }^{2}$ (relating to ${I}_{effect}^{2}$). Therefore, when ${\tau }^{2}$ (${I}_{study}^{2}$), or ${\sigma }^{2}$ (${I}_{effect}^{2}$), is close to zero, there will be little point fitting a moderator(s) at the level of studies, or effect sizes, respectively.

As in multiple regression, we can have multiple (multi-moderator) meta-regression, which can be written as:

11 $${z}_{i}={\beta }_{0}+\sum_{h=1}^{q}{\beta }_{h}{x}_{h\left[i\right]}+{u}_{j\left[i\right]}+{e}_{i}+{m}_{i},$$

where $\sum_{h=1}^{q}{\beta }_{h}{x}_{h\left[i\right]}$ denotes the sum of all the moderator effects, with *q* being the number of slopes (staring with *h* = 1). We note that *q* is not necessarily the number of moderators. This is because when we have a categorical moderator, which is common, with more than two levels (e.g., method A, B & C), the fixed effect part of the formula is ${\beta }_{0}+{\beta }_{1}{x}_{1}+{\beta }_{2}{x}_{2}$, where *x*_1_ and *x*_2_ are ‘dummy’ variables, which code whether the *i*th effect size belongs to, for example, method B or C, with ${\beta }_{1}$ and ${\beta }_{2}$ being contrasts between A and B and between A and C, respectively (for more explanations of dummy variables, see our tutorial page [https://itchyshin.github.io/Meta-analysis_tutorial/]; also see [67, 68]). Traditionally, researchers conduct separate meta-analyses per different groups (known as ‘sub-group analysis’), but we prefer a meta-regression approach with a categorical variable, which is statistically more powerful [40]. Also, importantly, what can be used as a moderator(s) is very flexible, including, for example, individual/plot characteristics (e.g., age, location), environmental factors (e.g., temperature), methodological differences between studies (e.g., randomization), and bibliometric information (e.g., publication year; see more in the section ‘Checking for publication bias and robustness’). Note that moderators should be decided and listed a priori in the meta-analysis plan (i.e., a review protocol or pre-registration).

As with meta-analysis, the *Q*-test (*Q*_*m*_ or *Q*_*moderator*_) is often used to test the significance of the moderator(s). To complement this test, we can also quantify variance explained by the moderator(s) using *R*^2^. We can define *R*^2^ using Eq. (11) as:

12 $${R}^{2}=\frac{{f}^{2}}{{f}^{2}+{\tau }^{2}+{\sigma }^{2}},$$

13 $${f}^{2}={\text{Var}}\left(\sum_{h=1}^{q}{\beta }_{h}{x}_{h\left[i\right]}\right),$$

where *R*^2^ is known as marginal *R*^2^ (sensu [69, 70]; cf. [71]), ${f}^{2}$ is the variance due to the moderator(s), and ${(f}^{2}+{\tau }^{2}+{\sigma }^{2})$ here equals to $({\tau }^{2}+{\sigma }^{2})$ in Eq. 7, as ${f}^{2}$ ‘absorbs’ variance from ${\tau }^{2}$ and/or ${\sigma }^{2}$. We can compare the similarities and differences in Fig. 3B where we denote a part of ${f}^{2}$ originating from ${\tau }^{2}$ as ${f}_{study}^{2}$ while ${\sigma }^{2}$ as ${f}_{effect}^{2}$. In a multiple meta-regression model, we often want to find a model with the ‘best’ or an adequate set of predictors (i.e., moderators). *R*^2^ can potentially help such a model selection process. Yet, methods based on information criteria (such as Akaike information criterion, AIC) may be preferable. Although model selection based on the information criteria is beyond the scope of the paper, we refer the reader to relevant articles (e.g., [72, 73]), and we show an example of this procedure in our online tutorial (https://itchyshin.github.io/Meta-analysis_tutorial/).

### Notes on visualisation and interpretation

**Fig. 4:**
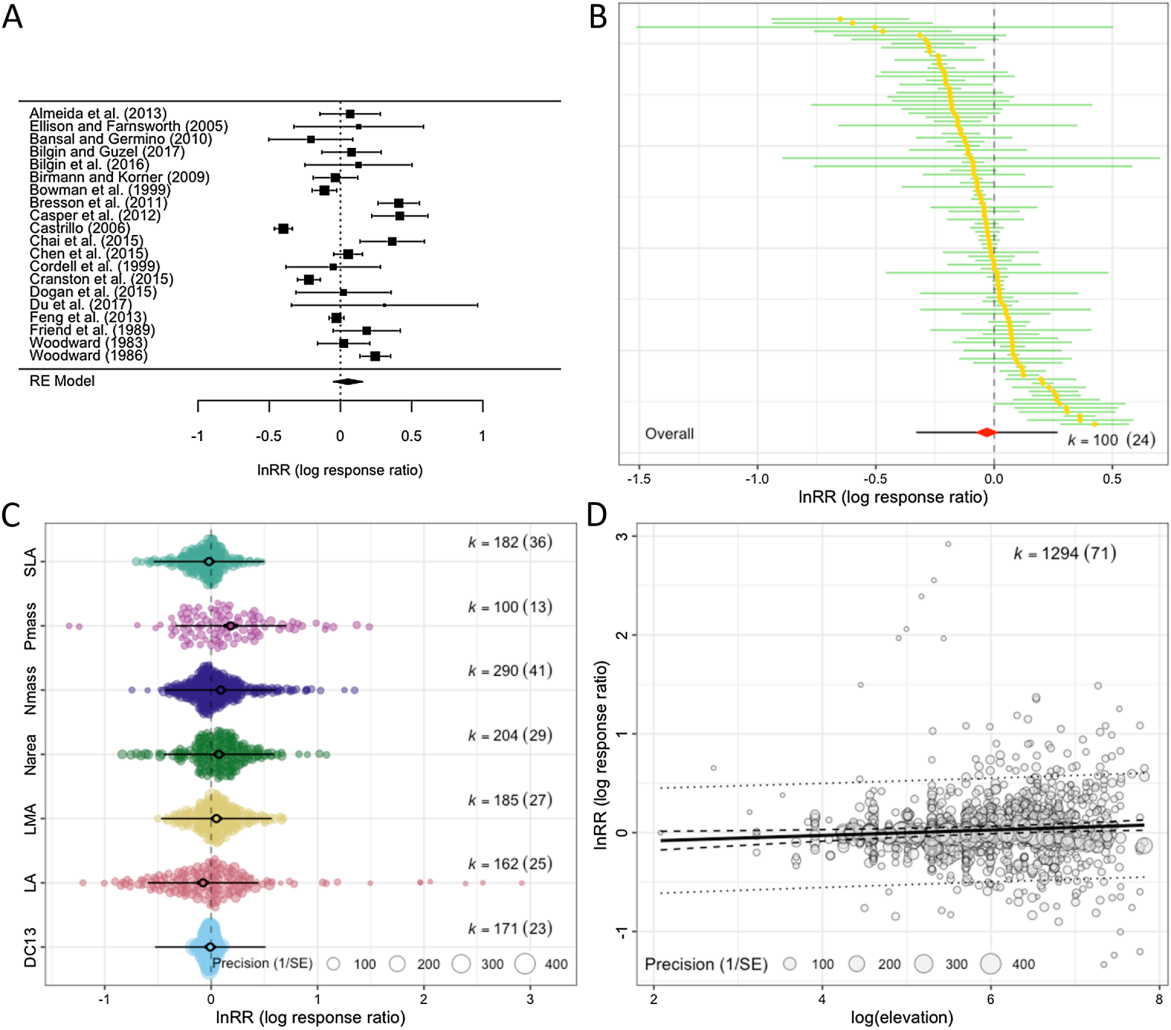
Different types of plots useful for a meta-analysis using data from Midolo et al. [133]: **A** a typical forest plot with the overall mean shown as a diamond at the bottom (20 effect sizes from 20 studies are used), **B** a caterpillar plot (100 effect sizes from 24 studies are used), **C** an orchard plot of categorical moderator with seven levels (all effect sizes are used), and **D** a bubble plot of a continuous moderator. Note that the first two only show confidence intervals, while the latter two also show prediction intervals (see the text for more details)

Visualization and interpretation of results is an essential part of a meta-analysis [74, 75]. Traditionally, a forest plot is used to display the values and 95% of confidence intervals (CIs) for each effect size and the overall effect and its 95% CI (the diamond symbol is often used, as shown in Fig. 4A). More recently, adding a 95% prediction interval (PI) to the overall estimate has been strongly recommended because 95% PIs show a predicted range of values in which an effect size from a new study would fall, assuming there is no sampling error [76]. Here, we think that examining the formulas for 95% CIs and PIs for the overall mean (from Eq. 3) is illuminating:

14 $$95\mathrm{\%}{\text{CI}}={\beta }_{0}\pm {t}_{df\left[\alpha =0.05\right]}se\left({\beta }_{0}\right),$$

15 $$95\mathrm{\%}{\text{PI}}={\beta }_{0}\pm {t}_{df\left[\alpha =0.05\right]}\sqrt{s{e}^{2}\left({\beta }_{0}\right)+{\tau }^{2}+{\sigma }^{2}},$$

where ${t}_{df\left[\alpha =0.05\right]}$ denotes the *t* value with the degree of freedom, *df*, at 97.5 percentile (or $\alpha =0.05$) and other notations are as above. In a meta-analysis, it has been conventional to use *z* value 1.96 instead of ${t}_{df\left[\alpha =0.05\right]}$, but simulation studies have shown the use of *t* value over *z* value reduces Type 1 errors under many scenarios and, therefore, is recommended (e.g., [13, 77]). Also, it is interesting to note that by plotting 95% PIs, we can visualize heterogeneity as Eq. 15 includes ${\tau }^{2}$ and ${\sigma }^{2}$.

A ‘forest’ plot can become quickly illegible as the number of studies (effect sizes) becomes large, so other methods of visualizing the distribution of effect sizes have been suggested. Some suggested to present a ‘caterpillar’ plot, which is a version of the forest plot, instead (Fig. 4B; e.g., [78]). We here recommend an ‘orchard’ plot, as it can present results across different groups (or a result of meta-regression with a categorical variable), as shown in Fig. 4C [78]. For visualization of a continuous variable, we suggest what is called a ‘bubble’ plot, shown in Fig. 4D. Visualization not only helps us interpret meta-analytic results, but can also help to identify something we may not see from statistical results, such as influential data points and outliers that could threaten the robustness of our results.

## Checking for publication bias and robustness

### Detecting and correcting for publication bias

**Fig. 5:**
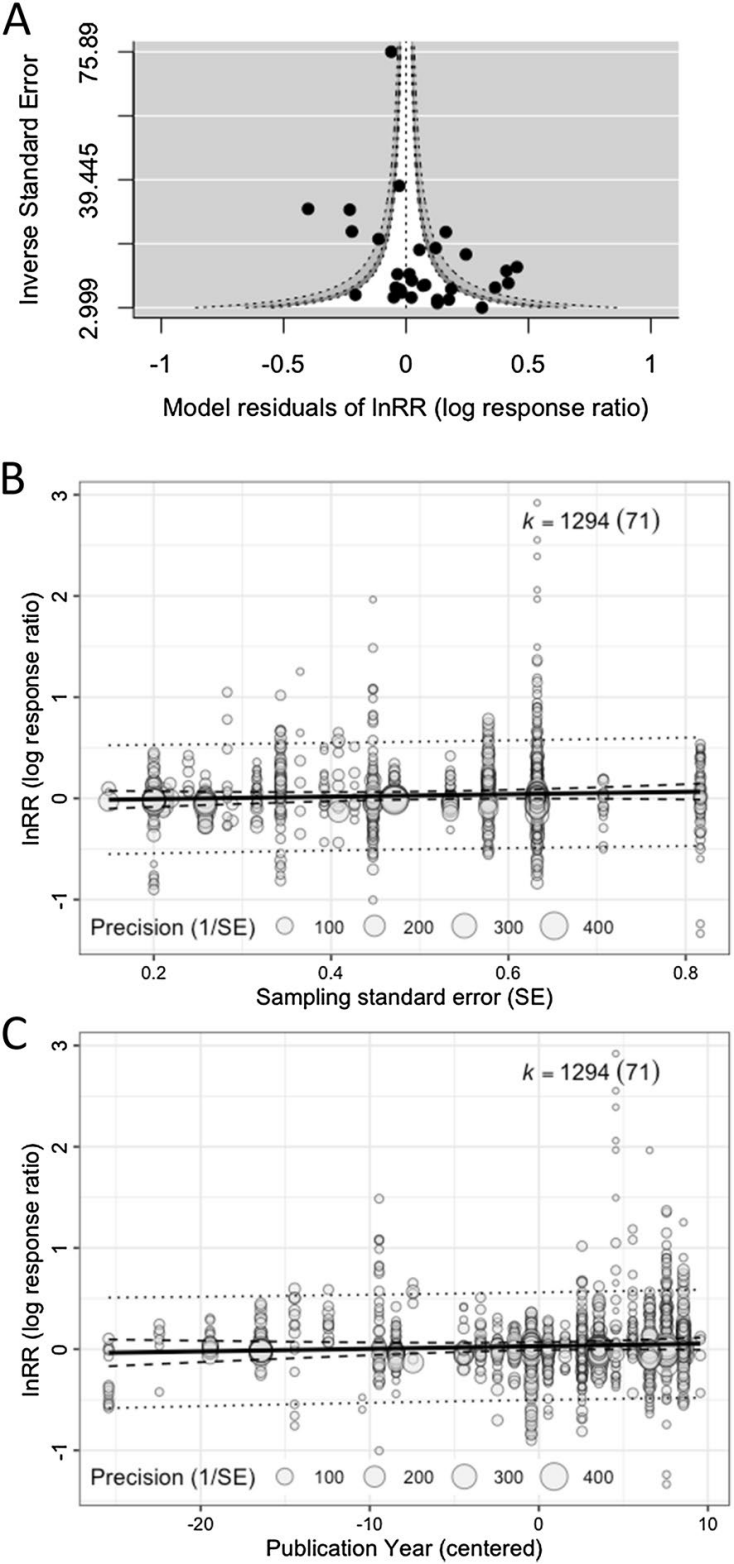
Different types of plots for publication bias tests: **A** a funnel plot using model residuals, showing a funnel (white) that shows the region of statistical non-significance (30 effect sizes from 30 studies are used; note that we used the inverse of standard errors for the *y*-axis, but for some effect sizes, sample size or ‘effective’ sample size may be more appropriate), **B** a bubble plot visualising a multilevel meta-regression that tests for the small study effect (note that the slope was non-significant: *b* = 0.120, 95% CI = [− 0.095, 0.334]; all effect sizes are used), and **C** a bubble plot visualising a multilevel meta-regression that tests for the decline effect (the slope was non-significant: *b* = 0.003, 95%CI = [− 0.002, 0.008])

Checking for and adjusting for any publication bias is necessary to ensure the validity of meta-analytic inferences [79]. However, our survey showed almost half of the environmental meta-analyses (46.6%; 34 out of 73 studies; Additional file 1) neither tested for nor corrected for publication bias (cf. [14–16]). The most popular methods used were: (1) graphical tests using funnel plots (26 studies; 35.6%), (2) regression-based tests such as Egger regression (18 studies; 24.7%), (3) Fail-safe number tests (12 studies; 16.4%), and (4) trim-and-fill tests (10 studies; 13.7%). We recently showed that these methods are unsuitable for datasets with non-independent effect sizes, with the exception of funnel plots [80] (for an example of funnel plots, see Fig. 5A). This is because these methods cannot deal with non-independence in the same way as the fixed-effect and random-effects models. Here, we only introduce a two-step method for multilevel models that can both detect and correct for publication bias [80] (originally proposed by [81, 82]), more specifically, the “small study effect” where an effect size value from a small-sample-sized study can be much larger in magnitude than a ‘true’ effect [83, 84]. This method is a simple extension of Egger’s regression [85], which can be easily implemented by using Eq. 10:

16 $${z}_{i}={\beta }_{0}+{\beta }_{1}\sqrt{\frac{1}{{\widetilde{n}}_{i}}}+{u}_{j\left[i\right]}+{e}_{i}+{m}_{i},$$

17 $${z}_{i}={\beta }_{0}+{\beta }_{1}\left(\frac{1}{{\widetilde{n}}_{i}}\right)+{u}_{j\left[i\right]}+{e}_{i}+{m}_{i},$$

where ${\widetilde{n}}_{i}$ is known as effective sample size; for *Zr* and proportion it is just *n*_*i*_, and for SMD and lnRR, it is ${n}_{iC}{n}_{iT}/\left({n}_{iC}+{n}_{iT}\right)$, as in Table 2. When ${\beta }_{1}$ is significant, we conclude there exists a small-study effect (in terms of a funnel plot, this is equivalent to significant funnel asymmetry). Then, we fit Eq. 17 and we look at the intercept ${\beta }_{0}$, which will be a bias-corrected overall estimate [note that ${\beta }_{0}$ in Eq. (16) provides less accurate estimates when non-zero overall effects exist [81, 82]; Fig. 5B]. An intuitive explanation of why ${\beta }_{0}$ (Eq. 17) is the ‘bias-corrected’ estimate is that the intercept represents $1/\widetilde{{n}_{i}}=0$ (or $\widetilde{{n}_{i}}=\infty$); in other words, ${\beta }_{0}$ is the estimate of the overall effect when we have a very large (infinite) sample size. Of note, appropriate bias correction requires a selection-mode-based approach although such an approach is yet to be available for multilevel meta-analytic models [80].

Conveniently, this proposed framework can be extended to test for another type of publication bias, known as time-lag bias, or the decline effect, where effect sizes tend to get closer to zero over time, as larger or statistically significant effects are published more quickly than smaller or non-statistically significant effects [86, 87]. Again, a decline effect can be statistically tested by adding year to Eq. (3):

18 $${z}_{i}={\beta }_{0}+{\beta }_{1}c\left(yea{r}_{j\left[i\right]}\right)+{u}_{j\left[i\right]}+{e}_{i}+{m}_{i},$$

where $c\left(yea{r}_{j\left[i\right]}\right)$ is the mean-centred publication year of a particular study (study *j* and effect size *i*); this centring makes the intercept ${\beta }_{0}$ meaningful, representing the overall effect estimate at the mean value of publication years (see [68]). When the slope is significantly different from 0, we deem that we have a decline effect (or time-lag bias; Fig. 5C).

However, there may be some confounding moderators, which need to be modelled together. Indeed, Egger’s regression (Eqs. 16 and 17) is known to detect the funnel asymmetry when there is little heterogeneity; this means that we need to model $\sqrt{1/{\widetilde{n}}_{i}}$ with other moderators that account for heterogeneity. Given this, we probably should use a multiple meta-regression model, as below:

19 $${z}_{i}={\beta }_{0}+{\beta }_{1}\sqrt{\frac{1}{{\widetilde{n}}_{i}}}+{\beta }_{2}c\left(yea{r}_{j\left[i\right]}\right)+\sum_{h=3}^{q}{\beta }_{h}{x}_{h\left[i\right]}+{u}_{j\left[i\right]}+{e}_{i}+{m}_{i},$$

where $\sum_{h=3}^{q}{\beta }_{h}{x}_{h\left[i\right]}$ is the sum of the other moderator effects apart from the small-study effect and decline effect, and other notations are as above (for more details see [80]). We need to carefully consider which moderators should go into Eq. 19 (e.g., fitting all moderators or using an AIC-based model selection method; see [72, 73]). Of relevance, when running complex models, some model parameters cannot be estimated well, or they are not ‘identifiable’ [88]. This is especially so for variance components (random-effect part) rather than regression coeffects (fixed-effect part). Therefore, it is advisable to check whether model parameters are all identifiable, which can be checked using the *profile* function in *metafor* (for an example, see our tutorial webpage [https://itchyshin.github.io/Meta-analysis_tutorial/]).

### Conducting sensitivity analysis and critical appraisal

Sensitivity analysis explores the robustness of meta-analytic results by running a different set of analyses from the original analysis, and comparing the results (note that some consider publication bias tests a part of sensitivity analysis; [11]). For example, we might be interested in assessing how robust results are to the presence of influential studies, to the choice of method for addressing non-independence, or weighting effect sizes. Unfortunately, in our survey, only 37% of environmental meta-analyses (27 out of 73) conducted sensitivity analysis (Additional file 1). There are two general and interrelated ways to conduct sensitivity analyses [73, 89, 90]. The first one is to take out influential studies (e.g., outliers) and re-run meta-analytic and meta-regression models. We can also systematically take each effect size out and run a series of meta-analytic models to see whether any resulting overall effect estimates are different from others; this method is known as ‘leave-one-out’, which is considered less subjective and thus recommended.

The second way of approaching sensitivity analysis is known as subset analysis, where a certain group of effect sizes (studies) will be excluded to re-run the models without this group of effect sizes. For example, one may want to run an analysis without studies that did not randomize samples. Yet, as mentioned earlier, we recommend using meta-regression (Eq. 13) with a categorical variable of randomization status (‘randomized’ or ‘not randomized’), to statistically test for an influence of moderators. It is important to note that such tests for risk of bias (or study quality) can be considered as a way of quantitatively evaluating the importance of study features that were noted at the stage of critical appraisal, which is an essential part of any systematic review (see [11, 91]). In other words, we can use meta-regression or subset analysis to quantitatively conduct critical appraisal using (study-level) moderators that code, for example, blinding, randomization, and selective reporting. Despite the importance of critical appraisal ([91]), only 4 of 73 environmental meta-analyses (5.6%) in our survey assessed the risk of bias in each study included in a meta-analysis (i.e., evaluating a primary study in terms of the internal validity of study design and reporting; Additional file 1). We emphasize that critically appraising each paper or checking them for risk of bias is an extremely important topic. Also, critical appraisal is not restricted to quantitative synthesis. Therefore, we do not cover any further in this paper for more, see [92, 93]).

### Notes on transparent reporting and open archiving

For environmental systematic reviews and maps, there are reporting guidelines called RepOrting standards for Systematic Evidence Syntheses in environmental research, ROSES [94] and synthesis assessment checklist, the Collaboration for Environmental Evidence Synthesis Appraisal Tool (CEESAT; [95]). However, these guidelines are somewhat limited in terms of reporting quantitative synthesis because they cover only a few core items. These two guidelines are complemented by the Preferred Reporting Items for Systematic Reviews and Meta-Analyses for Ecology and Evolutionary Biology (PRISMA-EcoEvo; [96]; cf. [97, 98]), which provides an extended set of reporting items covering what we have described above. Items 20–24 from PRISMA-EcoEvo are most relevant: these items outline what should be reported in the Methods section: (i) sample sizes and study characteristics, (ii) meta-analysis, (iii) heterogeneity, (iv) meta-regression and (v) outcomes of publication bias and sensitivity analysis (see Table 4). Our survey, as well as earlier surveys, suggest there is a large room for improvement in the current practice ([14–16]). Incidentally, the orchard plot is well aligned with Item 20, as this plot type shows both the number of effect sizes and studies for different groups (Fig. 4C). Further, our survey of environmental meta-analyses highlighted the poor standards of data openness (with 24 studies sharing data: 32.9%) and code sharing (7 studies: 29.2%; Additional file 1). Environmental scientists must archive their data as well as their analysis code in accordance with the FAIR principles (Findable, Accessible, Interoperable, and Reusable [99]) using dedicated depositories such as Dryad, FigShare, Open Science Framework (OSF), Zenodo or others (cf. [100, 101]), preferably not on publisher’s webpages (as paywall may block access). However, archiving itself is not enough; data requires metadata (detailed descriptions) and the code needs to also be FAIR [102, 103].

**Table 4:** Items relevant to reporting results for a meta-analysis from the Preferred Reporting Items for Systematic reviews and Meta-Analysis for Ecology and Evolutionary Biology (PRISMA-EcoEvo; [96])

Item	Description
20: Sample sizes and study characteristics	“Report the number of studies and effect size for data included in meta-analyses and subsets of data included in meta-regressions. Provide a summary of kye characteristics for reported outcomes (either in text or figures; e.g., one quarter of effect sizes reported for vertebrates and the rest invertebrates) and their limitations (e.g., collinearity and overlaps between moderators), including characteristics related individual study quality (risk of bias).”
21: Meta-analysis	“Provide a quantitative synthesis of results across studies, including estimates for the main effect size, with confidence/credible intervals.”
22: Heterogeneity	“Report indicators of heterogeneity in the estimated effect (e.g. *I*^2^, *tau*^2^ and other variance components).”
23: Meta-regression	“Provide estimates of meta-regression slopes (i.e. regression coefficients) for all variables that were assessed for their contribution to heterogeneity. Include confidence/credible intervals, and report interactions if they were included. Describe outcomes from model selection, if done (e.g. *R*^2^ and AIC).”
24: Outcomes of publication bias and sensitivity analysis	“Provide results for the assessments of the risks of bias (e.g. Egger’s regression, funnel plots) and robustness of the review’s results (e.g. subgroup analyses, meta-regression of study quality, results from alternative methods of analysis, and temporal trends)”

## Other relevant and advanced issues

### Scale dependence

The issue of scale dependence is a unique yet widespread problem in environmental sciences (see [7, 104]); our literature survey indicated three quarters of the environmental meta-analyses (56 out of 73 studies) have inferences that are potentially vulnerable to scale-dependence [105]. For example, studies that set out to compare group means in biodiversity measures, such as species richness, can vary as a function of the scale (size) of the sampling unit. When the unit of replication is a plot (not an individual animal or plant), the aerial size of a plot (e.g., 100 cm^2^ or 1 km^2^) will affect both the precision and accuracy of effect size estimates (e.g., lnRR and SMD). In general, a study with larger plots might have more accurately estimated species richness differences, but less precisely than a study with smaller plots and greater replication. Lower replication means that our sampling variance estimates are likely to be misestimated, and the study with larger plots will generally have less weight than the study with smaller plots, due to higher sampling variance. Inaccurate variance estimates in little-replicated ecological studies are known to cause an accumulating bias in precision-weighted meta-analysis, requiring correction [43]. To assess the potential for scale-dependence, it is recommended that analysts test for possible covariation among plot size, replication, variances, and effect sizes [104]. If detected, analysts should use an effect size measure that is less sensitive to scale dependence (lnRR), and could use the size of a plot as a moderator in meta-regression, or alternatively, they consider running an unweighted model ([7]; note that only 12%, 9 out of 73 studies, accounted for sampling area in some way; Additional file 1).

### Missing data

In many fields, meta-analytic data almost always encompass missing values see [106–108]. Broadly, we have two types of missing data in meta-analyses [109, 110]: (1) missing data in standard deviations or sample sizes, associated with means, preventing effect size calculations (Table 2), and (2) missing data in moderators. There are several solutions for both types. The best, and first to try, should be contacting the authors. If this fails, we can potentially ‘impute’ missing data. Single imputation methods using the strong correlation between standard deviation and mean values (known as mean–variance relationship) are available, although single imputation can lead to Type I error [106, 107] (see also [43]) because we do not model the uncertainty of imputation itself. Contrastingly, multiple imputation, which creates multiple versions of imputed datasets, incorporates such uncertainty. Indeed, multiple imputation is a preferred and proven solution for missing data in effect sizes and moderators [109, 110]. Yet, correct implementation can be challenging (see [110]). What we require now is an automated pipeline of merging meta-analysis and multiple imputation, which accounts for imputation uncertainty, although it may be challenging for complex meta-analytic models. Fortunately, however, for lnRR, there is a series of new methods that can perform better than the conventional method and which can deal with missing SDs [44]; note that these methods do not deal with missing moderators. Therefore, where applicable, we recommend these new methods, until an easy-to-implement multiple imputation workflow arrives.

### Complex non-independence

Above, we have only dealt with the model that includes study identities as a clustering/grouping (random) factor. However, many datasets are more complex, with potentially more clustering variables in addition to the study identity. It is certainly possible that an environmental meta-analysis contains data from multiple species. Such a situation creates an interesting dependence among effect sizes from different species, known as phylogenetic relatedness, where closely related species are more likely to be similar in effect sizes compared to distantly related ones (e.g., mice *vs.* rats and mice *vs.* sparrows). Our multilevel model framework is flexible and can accommodate phylogenetic relatedness. A phylogenetic multilevel meta-analytic model can be written as [40, 111, 112]:

20 $${z}_{i}={\beta }_{0}+{a}_{k\left[i\right]}+{s}_{k\left[i\right]}+{u}_{j\left[i\right]}+{e}_{i}+{m}_{i},$$

$${a}_{k}\sim \mathrm{N}\left(0,{\omega }^{2}{\text{A}}\right), {s}_{k}\sim \mathrm{N}\left(0,{\gamma }^{2}\right), {u}_{j}\sim \mathrm{N}\left(0,{\tau }^{2}\right), {e}_{i}\sim \mathrm{N}\left(0,{\sigma }^{2}\right),\, \& \ {m}_{i}\sim \mathrm{N}\left(0,{v}_{i}\right),$$

where ${a}_{k\left[i\right]}$ is the phylogenetic (species) effect for the *k*th species (effect size *i*; *N*_*effect*_ (*i* = 1, 2,…, *N*_*effect*_) > *N*_*study*_ (*j* = 1, 2,…, *N*_*study*_) > *N*_*species*_ (*k* = 1, 2,…, *N*_*species*_)), normally distributed with ${\omega }^{2}{\text{A}}$ where is the phylogenetic variance and **A** is a correlation matrix coding how close each species are to each other and ${\omega }^{2}$ is the phylogenetic variance, ${s}_{k\left[i\right]}$ is the non-phylogenetic (species) effect for the *k*th species (effect size *i*), normally distributed with the variance of ${\gamma }^{2}$ (the non-phylogenetic variance), and other notations are as above. It is important to realize that **A** explicitly models relatedness among species, and we do need to provide this correlation matrix, using a distance relationship usually derived from a molecular-based phylogenetic tree (for more details, see [40, 111, 112]). Some may think that the non-phylogenetic term (${s}_{k\left[i\right]}$) is unnecessary or redundant because ${s}_{k\left[i\right]}$ and the phylogenetic term (${a}_{k\left[i\right]}$) are both modelling variance at the species level. However, a simulation recently demonstrated that failing to have the non-phylogenetic term (${s}_{k\left[i\right]}$) will often inflate the phylogenetic variance ${\omega }^{2}$, leading to an incorrect conclusion that there is a strong phylogenetic signal (as shown in [112]). The non-phylogenetic variance (${\gamma }^{2}$) arises from, for example, ecological similarities among species (herbivores *vs.* carnivores or arboreal *vs.* ground-living) not phylogeny [40].

Like phylogenetic relatedness, effect sizes arising from closer geographical locations are likely to be more correlated [113]. Statistically, spatial correlation can be also modelled in a manner analogous to phylogenetic relatedness (i.e., rather than a phylogenetic correlation matrix, **A**, we fit a spatial correlation matrix). For example, Maire and colleagues [114] used a meta-analytic model with spatial autocorrelation to investigate the temporal trends of fish communities in the network of rivers in France. We note that a similar argument can be made for temporal correlation, but in many cases, temporal correlations could be dealt with, albeit less accurately, as a special case of ‘shared measurements’, as in Fig. 2. An important idea to take away is that one can model different, if not all, types of non-independence as the random factor(s) in a multilevel model.

### Advanced techniques

Here we touch upon five advanced meta-analytic techniques with potential utility for environmental sciences, providing relevant references so that interested readers can obtain more information on these advanced topics. The first one is the meta-analysis of magnitudes, or absolute values (effect sizes), where researchers may be interested in deviations from 0, rather than the directionality of the effect [115]. For example, Cohen and colleagues [116] investigated absolute values of phenological responses, as they were concerned with the magnitudes of changes in phenology rather than directionality.

The second method is the meta-analysis of interaction where our focus is on synthesizing the interaction effect of, usually, 2 × 2 factorial design (e.g., the effect of two simultaneous environmental stressors [54, 117, 118]; see also [119]). Recently, Siviter and colleagues [120] showed that agrochemicals interact synergistically (i.e., non-additively) to increase the mortality of bees; that is, two agrochemicals together caused more mortality than the sum of mortalities of each chemical.

Third, network meta-analysis has been heavily used in medical sciences; network meta-analysis usually compares different treatments in relation to placebo and ranks these treatments in terms of effectiveness [121]. The very first ‘environmental’ network meta-analysis, as far as we know, investigated the effectives of ecosystem services among different land types [122].

Fourth, a multivariate meta-analysis is where one can model two or more different types of effect sizes with the estimation of pair-wise correlations between different effect sizes. The benefit of such an approach is known as the ‘borrowing of strength’, where the error of fixed effects (moderators; e.g., *b*_0_ and *b*_1_) can be reduced when different types of effect sizes are correlated (i.e., *se*(*b*_0_) and *se*(*b*_1_) can be smaller [123]) For example, it is possible for lnRR (differences in mean) and lnVR (differences in SDs) to be modelled together (cf. [124]).

Fifth, as with network meta-analysis, there has been a surge in the use of ‘individual participants data’, called ‘IPD meta-analysis’, in medical sciences [125, 126]. The idea of IPD meta-analysis is simple—rather than using summary statistics reported in papers (sample means and variances), we directly use raw data from all studies. We can either model raw data using one complex multilevel (hierarchical) model (one-step method) or calculate statistics for each study and use a meta-analysis (two-step method; note that both methods will usually give the same results). Study-level random effects can be incorporated to allow the response variable of interest to vary among studies, and overall effects correspond to fixed, population-level estimates. The use of IPD or ‘full-data analyses’ has also surged in ecology, aided by open-science policies that encourage the archival of raw data alongside articles, and initiatives that synthesise raw data (e.g., PREDICTS [127], BioTime [128]). In health disciplines, such meta-analyses are considered the ‘gold standard’ [129], owing to their potential for resolving issues regarding study-specific designs and confounding variation, and it is unclear whether and how they might resolve issues such as scale dependence in environmental meta-analyses [104, 130].

## Conclusions

In this article, we have attempted to describe the most practical ways to conduct quantitative synthesis, including meta-analysis, meta-regression, and publication bias tests. In addition, we have shown that there is much to be improved in terms of meta-analytic practice and reporting via a survey of 73 recent environmental meta-analyses. Such improvements are urgently required, especially given the potential influence that environmental meta-analyses can have on policies and decision-making [8]. So often, meta-analysts have called for better reporting of primary research (e.g. [131, 132]), and now this is the time to raise the standards of reporting in meta-analyses. We hope our contribution will help to catalyse a turning point for better practice in quantitative synthesis in environmental sciences. We remind the reader most of what is described is implemented in the *R* environment on our tutorial webpage and researchers can readily use the proposed models and techniques (https://itchyshin.github.io/Meta-analysis_tutorial/). Finally, meta-analytic techniques are always developing and improving. It is certainly possible that in the future, our proposed models and related methods will become dated, just as the traditional fixed-effect and random-effects models already are. Therefore, we must endeavour to be open-minded to new ways of doing quantitative research synthesis in environmental sciences.

## Supplementary Information

**Additional file 1:** The survey of meta-analyses in environmnetal sciences.

**Additional file 2:** The hands-on R tutorial.

## Data Availability

All data and material are provided as additional files.

## Declarations

### Contest for publication

The authors provide consent for publication.

## References

[1] Higgins JP, Thomas JE, Chandler JE, Cumpston ME, Li TE, Page MJ, Welch VA. Cochrane handbook for systematic reviews of interventions. 2nd ed. Chichester: Wikey; 2019.

[2] Cooper HM, Hedges LV, Valentine JC. **The handbook of research synthesis and meta-analysis**. 3rd ed. New York: Russell Sage Foundation; 2019.

[3] Schmid CH, Stijnen TE, White IE. Handbook of meta-analysis. 1st ed. Boca Ranton: CRC; 2021.

[4] Vetter D, Rucker G, Storch I. Meta-analysis: a need for well-defined usage in ecology and conservation biology. Ecosphere. 2013;4(6):1. 10.1890/ES13-00062.1

[5] Koricheva J, Gurevitch J, Mengersen K, editors. Handbook of meta-analysis in ecology and evolution. Princeton: Princeton Univesity Press; 2017.

[6] Gurevitch J, Koricheva J, Nakagawa S, Stewart G. Meta-analysis and the science of research synthesis. Nature. 2018;555(7695):175–82. 29517004 10.1038/nature25753

[7] Spake R, Doncaster CP. Use of meta-analysis in forest biodiversity research: key challenges and considerations. Forest Ecol Manag. 2017;400:429–37. 10.1016/j.foreco.2017.05.059

[8] Bilotta GS, Milner AM, Boyd I. On the use of systematic reviews to inform environmental policies. Environ Sci Policy. 2014;42:67–77. 10.1016/j.envsci.2014.05.010

[9] Hedges LV, Vevea JL. Fixed- and random-effects models in meta-analysis. Psychol Methods. 1998;3(4):486–504. 10.1037/1082-989X.3.4.486

[10] Borenstein M, Hedges LV, Higgins JPT, Rothstein H. Introduction to meta-analysis. 2nd ed. Chichester: Wiley; 2021.

[11] Noble DWA, Lagisz M, Odea RE, Nakagawa S. Nonindependence and sensitivity analyses in ecological and evolutionary meta-analyses. Mol Ecol. 2017;26(9):2410–25. 28133832 10.1111/mec.14031

[12] Nakagawa S, Noble DWA, Senior AM, Lagisz M. Meta-evaluation of meta-analysis: ten appraisal questions for biologists. Bmc Biol. 2017;15:1. 28257642 10.1186/s12915-017-0357-7 PMC5336618

[13] Nakagawa S, Senior AM, Viechtbauer W, Noble DWA. An assessment of statistical methods for nonindependent data in ecological meta-analyses: comment. Ecology. 2022;103(1): e03490. 34292593 10.1002/ecy.3490

[14] Romanelli JP, Meli P, Naves RP, Alves MC, Rodrigues RR. Reliability of evidence-review methods in restoration ecology. Conserv Biol. 2021;35(1):142–54. 33347737 10.1111/cobi.13661

[15] Koricheva J, Gurevitch J. Uses and misuses of meta-analysis in plant ecology. J Ecol. 2014;102(4):828–44. 10.1111/1365-2745.12224

[16] O’Leary BC, Kvist K, Bayliss HR, Derroire G, Healey JR, Hughes K, Kleinschroth F, Sciberras M, Woodcock P, Pullin AS. The reliability of evidence review methodology in environmental science and conservation. Environ Sci Policy. 2016;64:75–82. 10.1016/j.envsci.2016.06.012

[17] Rosenthal R. The “file drawer problem” and tolerance for null results. Psychol Bull. 1979;86(3):638–41. 10.1037/0033-2909.86.3.638

[18] Nakagawa S, Lagisz M, Jennions MD, Koricheva J, Noble DWA, Parker TH, Sánchez-Tójar A, Yang Y, O’Dea RE. Methods for testing publication bias in ecological and evolutionary meta-analyses. Methods Ecol Evol. 2022;13(1):4–21. 10.1111/2041-210X.13724

[19] Cheung MWL. A guide to conducting a meta-analysis with non-independent effect sizes. Neuropsychol Rev. 2019;29(4):387–96. 31446547 10.1007/s11065-019-09415-6 PMC6892772

[20] Viechtbauer W. Conducting meta-analyses in R with the metafor package. J Stat Softw. 2010;36(3):1–48. 10.18637/jss.v036.i03

[21] Yang Y, Macleod M, Pan J, Lagisz M, Nakagawa S. Advanced methods and implementations for the meta-analyses of animal models: current practices and future recommendations. Neurosci Biobehav Rev. 2022. 10.1016/j.neubiorev.2022.105016:105016. 10.1016/j.neubiorev.2022.105016:105016 36566804

[22] Nakagawa S, Cuthill IC. Effect size, confidence interval and statistical significance: a practical guide for biologists. Biol Rev. 2007;82(4):591–605. 17944619 10.1111/j.1469-185X.2007.00027.x

[23] Hedges LV, Gurevitch J, Curtis PS. The meta-analysis of response ratios in experimental ecology. Ecology. 1999;80(4):1150–6. 10.1890/0012-9658(1999)080[1150:TMAORR]2.0.CO;2

[24] Friedrich JO, Adhikari NKJ, Beyene J. The ratio of means method as an alternative to mean differences for analyzing continuous outcome variables in meta-analysis: A simulation study. BMC Med Res Methodol. 2008;8:5. 18492289 10.1186/1471-2288-8-32 PMC2430201

[25] Hedges L, Olkin I. Statistical methods for meta-analysis. New York: Academic Press; 1985.

[26] Cohen J. Statistical power analysis for the beahvioral sciences. 2nd ed. Hillsdale: Lawrence Erlbaum; 1988.

[27] Senior AM, Viechtbauer W, Nakagawa S. Revisiting and expanding the meta-analysis of variation: the log coefficient of variation ratio. Res Synth Methods. 2020;11(4):553–67. 32431099 10.1002/jrsm.1423

[28] Nakagawa S, Poulin R, Mengersen K, Reinhold K, Engqvist L, Lagisz M, Senior AM. Meta-analysis of variation: ecological and evolutionary applications and beyond. Methods Ecol Evol. 2015;6(2):143–52. 10.1111/2041-210X.12309

[29] Knapp S, van der Heijden MGA. A global meta-analysis of yield stability in organic and conservation agriculture. Nat Commun. 2018;9:3632. 30194344 10.1038/s41467-018-05956-1 PMC6128901

[30] Porturas LD, Anneberg TJ, Cure AE, Wang SP, Althoff DM, Segraves KA. A meta-analysis of whole genome duplication and theeffects on flowering traits in plants. Am J Bot. 2019;106(3):469–76. 30901499 10.1002/ajb2.1258

[31] Janicke T, Morrow EH. Operational sex ratio predicts the opportunity and direction of sexual selection across animals. Ecol Lett. 2018;21(3):384–91. 29341415 10.1111/ele.12907

[32] Chamberlain R, Brunswick N, Siev J, McManus IC. Meta-analytic findings reveal lower means but higher variances in visuospatial ability in dyslexia. Brit J Psychol. 2018;109(4):897–916. 29938776 10.1111/bjop.12321

[33] O’Dea RE, Lagisz M, Jennions MD, Nakagawa S. Gender differences in individual variation in academic grades fail to fit expected patterns for STEM. Nat Commun. 2018;9:3777. 30254267 10.1038/s41467-018-06292-0 PMC6156605

[34] Brugger SP, Angelescu I, Abi-Dargham A, Mizrahi R, Shahrezaei V, Howes OD. Heterogeneity of striatal dopamine function in schizophrenia: meta-analysis of variance. Biol Psychiat. 2020;87(3):215–24. 31561858 10.1016/j.biopsych.2019.07.008

[35] Usui T, Macleod MR, McCann SK, Senior AM, Nakagawa S. Meta-analysis of variation suggests that embracing variability improves both replicability and generalizability in preclinical research. Plos Biol. 2021;19(5): e3001009. 34010281 10.1371/journal.pbio.3001009 PMC8168858

[36] Hoffmann AA, Merila J. Heritable variation and evolution under favourable and unfavourable conditions. Trends Ecol Evol. 1999;14(3):96–101. 10322508 10.1016/S0169-5347(99)01595-5

[37] Wood CW, Brodie ED 3rd. Environmental effects on the structure of the G-matrix. Evolution. 2015;69(11):2927–40. 26462609 10.1111/evo.12795

[38] Hillebrand H, Donohue I, Harpole WS, Hodapp D, Kucera M, Lewandowska AM, Merder J, Montoya JM, Freund JA. Thresholds for ecological responses to global change do not emerge from empirical data. Nat Ecol Evol. 2020;4(11):1502. 32807945 10.1038/s41559-020-1256-9 PMC7614041

[39] Yang YF, Hillebrand H, Lagisz M, Cleasby I, Nakagawa S. Low statistical power and overestimated anthropogenic impacts, exacerbated by publication bias, dominate field studies in global change biology. Global Change Biol. 2022;28(3):969–89. 10.1111/gcb.15972 PMC9299651 34736291

[40] Nakagawa S, Santos ESA. Methodological issues and advances in biological meta-analysis. Evol Ecol. 2012;26(5):1253–74. 10.1007/s10682-012-9555-5

[41] Bakbergenuly I, Hoaglin DC, Kulinskaya E. Estimation in meta-analyses of response ratios. BMC Med Res Methodol. 2020;20(1):1. 10.1186/s12874-020-01137-1 PMC7579974 33092521

[42] Bakbergenuly I, Hoaglin DC, Kulinskaya E. Estimation in meta-analyses of mean difference and standardized mean difference. Stat Med. 2020;39(2):171–91. 31709582 10.1002/sim.8422 PMC6916299

[43] Doncaster CP, Spake R. Correction for bias in meta-analysis of little-replicated studies. Methods Ecol Evol. 2018;9(3):634–44. 29938012 10.1111/2041-210X.12927 PMC5993351

[44] Nakagawa S, Noble DW, Lagisz M, Spake R, Viechtbauer W, Senior AM. A robust and readily implementable method for the meta-analysis of response ratios with and without missing standard deviations. Ecol Lett. 2023;26(2):232–44 36573275 10.1111/ele.14144 PMC10108319

[45] Hamman EA, Pappalardo P, Bence JR, Peacor SD, Osenberg CW. Bias in meta-analyses using Hedges’ d. Ecosphere. 2018;9(9): e02419. 10.1002/ecs2.2419

[46] Bakbergenuly I, Hoaglin DC, Kulinskaya E. On the Q statistic with constant weights for standardized mean difference. Brit J Math Stat Psy. 2022;75(3):444–65. 10.1111/bmsp.12263 35094381

[47] DerSimonian R, Kacker R. Random-effects model for meta-analysis of clinical trials: an update. Contemp Clin Trials. 2007;28(2):105–14. 16807131 10.1016/j.cct.2006.04.004

[48] Veroniki AA, Jackson D, Viechtbauer W, Bender R, Bowden J, Knapp G, Kuss O, Higgins JPT, Langan D, Salanti G. Methods to estimate the between-study variance and its uncertainty in meta-analysis. Res Synth Methods. 2016;7(1):55–79. 26332144 10.1002/jrsm.1164 PMC4950030

[49] Langan D, Higgins JPT, Simmonds M. Comparative performance of heterogeneity variance estimators in meta-analysis: a review of simulation studies. Res Synth Methods. 2017;8(2):181–98. 27060925 10.1002/jrsm.1198

[50] Panityakul T, Bumrungsup C, Knapp G. On estimating residual heterogeneity in random-effects meta-regression: a comparative study. J Stat Theory Appl. 2013;12(3):253–65. 10.2991/jsta.2013.12.3.4

[51] Bishop J, Nakagawa S. Quantifying crop pollinator dependence and its heterogeneity using multi-level meta-analysis. J Appl Ecol. 2021;58(5):1030–42. 10.1111/1365-2664.13830

[52] Cheung MWL. Modeling dependent effect sizes with three-level meta-analyses: a structural equation modeling approach. Psychol Methods. 2014;19(2):211–29. 23834422 10.1037/a0032968

[53] Bolker BM, Brooks ME, Clark CJ, Geange SW, Poulsen JR, Stevens MHH, White JSS. Generalized linear mixed models: a practical guide for ecology and evolution. Trends Ecol Evol. 2009;24(3):127–35. 19185386 10.1016/j.tree.2008.10.008

[54] Lajeunesse MJ. On the meta-analysis of response ratios for studies with correlated and multi-group designs. Ecology. 2011;92(11):2049–55. 22164829 10.1890/11-0423.1

[55] Gleser LJ, Olkin I. Stochastically dependent effect sizes. In: Cooper H, Hedges LV, Valentine JC, editors. The handbook of research synthesis and meta-analysis. New York: Russell Sage Foundation; 2009.

[56] Tipton E, Pustejovsky JE. Small-sample adjustments for tests of moderators and model fit using robust variance estimation in meta-regression. J Educ Behav Stat. 2015;40(6):604–34. 10.3102/1076998615606099

[57] Hedges LV, Tipton E, Johnson MC. Robust variance estimation in meta-regression with dependent effect size estimates (vol 1, pg 39, 2010). Res Synth Methods. 2010;1(2):164–5. 26056092 10.1002/jrsm.17

[58] Pustejovsky JE, Tipton E. Meta-analysis with robust variance estimation: expanding the range of working models. Prev Sci. 2021. 10.1007/s11121-021-01246-3. 10.1007/s11121-021-01246-3 33961175

[59] Cairns M, Prendergast LA. On ratio measures of heterogeneity for meta-analyses. Res Synth Methods. 2022;13(1):28–47. 34328266 10.1002/jrsm.1517

[60] Borenstein M, Higgins JPT, Hedges LV, Rothstein HR. Basics of meta-analysis: I2 is not an absolute measure of heterogeneity. Res Synth Methods. 2017;8(1):5–18. 28058794 10.1002/jrsm.1230

[61] Hoaglin DC. Practical challenges of I-2 as a measure of heterogeneity. Res Synth Methods. 2017;8(3):254–254. 28631294 10.1002/jrsm.1251

[62] Higgins JPT, Thompson SG. Quantifying heterogeneity in a meta-analysis. Stat Med. 2002;21(11):1539–58. 12111919 10.1002/sim.1186

[63] Higgins JPT, Thompson SG, Deeks JJ, Altman DG. Measuring inconsistency in meta-analyses. Brit Med J. 2003;327(7414):557–60. 12958120 10.1136/bmj.327.7414.557 PMC192859

[64] Xiong CJ, Miller JP, Morris JC. Measuring study-specific heterogeneity in meta-analysis: application to an antecedent biomarker study of Alzheimer’s disease. Stat Biopharm Res. 2010;2(3):300–9. 20703369 10.1198/sbr.2009.0067 PMC2919159

[65] Nakagawa S, Schielzeth H. Repeatability for Gaussian and non-Gaussian data: a practical guide for biologists. Biol Rev. 2010;85(4):935–56. 20569253 10.1111/j.1469-185X.2010.00141.x

[66] Senior AM, Grueber CE, Kamiya T, Lagisz M, O’Dwyer K, Santos ESA, Nakagawa S. Heterogeneity in ecological and evolutionary meta-analyses: its magnitude and implications. Ecology. 2016;97(12):3293–9. 27912008 10.1002/ecy.1591

[67] Gelman A, Hill J. Data analysis using regression and multilevel/hierarchical models. Cambridge: Cambridge University Press; 2007.

[68] Schielzeth H. Simple means to improve the interpretability of regression coefficients. Methods Ecol Evol. 2010;1(2):103–13. 10.1111/j.2041-210X.2010.00012.x

[69] Nakagawa S, Schielzeth H. A general and simple method for obtaining R2 from generalized linear mixed-effects models. Methods Ecol Evol. 2013;4(2):133–42. 10.1111/j.2041-210x.2012.00261.x

[70] Nakagawa S, Johnson PCD, Schielzeth H. The coefficient of determination R-2 and intra-class correlation coefficient from generalized linear mixed-effects models revisited and expanded. J R Soc Interface. 2017;14(134):20170213. 28904005 10.1098/rsif.2017.0213 PMC5636267

[71] Aloe AM, Becker BJ, Pigott TD. An alternative to R-2 for assessing linear models of effect size. Res Synth Methods. 2010;1(3–4):272–83. 26061471 10.1002/jrsm.23

[72] Cinar O, Umbanhowar J, Hoeksema JD, Viechtbauer W. Using information-theoretic approaches for model selection in meta-analysis. Res Synth Methods. 2021. 10.1002/jrsm.1489. 10.1002/jrsm.1489 PMC8359854 33932323

[73] Viechtbauer W. Model checking in meta-analysis. In: Schmid CH, Stijnen T, White IR, editors. Handbook of meta-analysis. Boca Raton: CRC; 2021.

[74] Anzures-Cabrera J, Higgins JPT. Graphical displays for meta-analysis: An overview with suggestions for practice. Res Synth Methods. 2010;1(1):66–80. 26056093 10.1002/jrsm.6

[75] Kossmeier M, Tran US, Voracek M. Charting the landscape of graphical displays for meta-analysis and systematic reviews: a comprehensive review, taxonomy, and feature analysis. Bmc Med Res Methodol. 2020;20(1):1. 10.1186/s12874-020-0911-9 PMC7006175 32028897

[76] Intout J, Ioannidis JPA, Rovers MM, Goeman JJ. Plea for routinely presenting prediction intervals in meta-analysis. BMJ Open. 2016;6(7): e010247. 10.1136/bmjopen-2015-010247 PMC4947751 27406637

[77] Moeyaert M, Ugille M, Beretvas SN, Ferron J, Bunuan R, Van den Noortgate W. Methods for dealing with multiple outcomes in meta-analysis a comparison between averaging effect sizes, robust variance estimation and multilevel meta-analysis. Int J Soc Res Methodol. 2017;20:559. 10.1080/13645579.2016.1252189

[78] Nakagawa S, Lagisz M, O’Dea RE, Rutkowska J, Yang YF, Noble DWA, Senior AM. The orchard plot: cultivating a forest plot for use in ecology, evolution, and beyond. Res Synth Methods. 2021;12(1):4–12. 32445243 10.1002/jrsm.1424

[79] Rothstein H, Sutton AJ, Borenstein M. Publication bias in meta-analysis : prevention, assessment and adjustments. Hoboken: Wiley; 2005.

[80] Nakagawa S, Lagisz M, Jennions MD, Koricheva J, Noble DWA, Parker TH, Sanchez-Tojar A, Yang YF, O’Dea RE. Methods for testing publication bias in ecological and evolutionary meta-analyses. Methods Ecol Evol. 2022;13(1):4–21. 10.1111/2041-210X.13724

[81] Stanley TD, Doucouliagos H. Meta-regression analysis in economics and business. New York: Routledge; 2012.

[82] Stanley TD, Doucouliagos H. Meta-regression approximations to reduce publication selection bias. Res Synth Methods. 2014;5(1):60–78. 26054026 10.1002/jrsm.1095

[83] Sterne JAC, Becker BJ, Egger M. The funnel plot. In: Rothstein H, Sutton AJ, Borenstein M, editors. Publication bias in meta-analysis: prevention, assessment and adjustments. Chichester: Wiley; 2005. p. 75–98.

[84] Sterne JAC, Sutton AJ, Ioannidis JPA, Terrin N, Jones DR, Lau J, Carpenter J, Rucker G, Harbord RM, Schmid CH, et al. Recommendations for examining and interpreting funnel plot asymmetry in meta-analyses of randomised controlled trials. Br Med J. 2011;343:4002. 10.1136/bmj.d4002 21784880

[85] Egger M, Smith GD, Schneider M, Minder C. Bias in meta-analysis detected by a simple, graphical test. Brit Med J. 1997;315(7109):629–34. 9310563 10.1136/bmj.315.7109.629 PMC2127453

[86] Jennions MD, Moller AP. Relationships fade with time: a meta-analysis of temporal trends in publication in ecology and evolution. P Roy Soc B-Biol Sci. 2002;269(1486):43–8. 10.1098/rspb.2001.1832 PMC1690867 11788035

[87] Koricheva J, Kulinskaya E. Temporal instability of evidence base: a threat to policy making? Trends Ecol Evol. 2019;34(10):895–902. 31196571 10.1016/j.tree.2019.05.006

[88] Raue A, Kreutz C, Maiwald T, Bachmann J, Schilling M, Klingmuller U, Timmer J. Structural and practical identifiability analysis of partially observed dynamical models by exploiting the profile likelihood. Bioinformatics. 2009;25(15):1923–9. 19505944 10.1093/bioinformatics/btp358

[89] Matsushima Y, Noma H, Yamada T, Furukawa TA. Influence diagnostics and outlier detection for meta-analysis of diagnostic test accuracy. Res Synth Methods. 2020;11(2):237–47. 31724796 10.1002/jrsm.1387

[90] Viechtbauer W, Cheung MWL. Outlier and influence diagnostics for meta-analysis. Res Synth Methods. 2010;1(2):112–25. 26061377 10.1002/jrsm.11

[91] Haddaway NR, Macura B. The role of reporting standards in producing robust literature reviews comment. Nat Clim Change. 2018;8(6):444–7. 10.1038/s41558-018-0180-3

[92] Frampton G, Whaley P, Bennett M, Bilotta G, Dorne JLCM, Eales J, James K, Kohl C, Land M, Livoreil B, et al. Principles and framework for assessing the risk of bias for studies included in comparative quantitative environmental systematic reviews. Environ Evid. 2022;11(1):12. 10.1186/s13750-022-00264-0 PMC10805236 38264537

[93] Stanhope J, Weinstein P. Critical appraisal in ecology: what tools are available, and what is being used in systematic reviews? Res Synth Methods. 2022. 10.1002/jrsm.1609. 10.1002/jrsm.1609 36303454

[94] Haddaway NR, Macura B, Whaley P, Pullin AS. ROSES RepOrting standards for systematic evidence syntheses: pro forma, flow-diagram and descriptive summary of the plan and conduct of environmental systematic reviews and systematic maps. Environ Evid. 2018;7(1):1. 10.1186/s13750-018-0121-7

[95] Woodcock P, Pullin AS, Kaiser MJ. Evaluating and improving the reliability of evidence syntheses in conservation and environmental science: a methodology. Biol Conserv. 2014;176:54–62. 10.1016/j.biocon.2014.04.020

[96] O’Dea RE, Lagisz M, Jennions MD, Koricheva J, Noble DWA, Parker TH, Gurevitch J, Page MJ, Stewart G, Moher D, et al. Preferred reporting items for systematic reviews and meta-analyses in ecology and evolutionary biology: a PRISMA extension. Biol Rev. 2021;96(5):1695–722. 33960637 10.1111/brv.12721 PMC8518748

[97] Moher D, Liberati A, Tetzlaff J, Altman DG. Preferred reporting items for systematic reviews and meta-analyses: the PRISMA statement. Plos Med. 2009;6(7):e1000097. 19621072 10.1371/journal.pmed.1000097 PMC2707599

[98] Page MJ, McKenzie JE, Bossuyt PM, Boutron I, Hoffmann TC, Mulrow CD, Shamseer L, Tetzlaff JM, Akl EA, Brennan SE, et al. The PRISMA 2020 statement: an updated guideline for reporting systematic reviews. Plos Med. 2021;18(3): e1003583. 33780438 10.1371/journal.pmed.1003583 PMC8007028

[99] Wilkinson MD, Dumontier M, Aalbersberg IJ, Appleton G, Axton M, Baak A, Blomberg N, Boiten JW, Santos LBD, Bourne PE, et al. Comment: the FAIR guiding principles for scientific data management and stewardship. Sci Data. 2016;3: 160018. 26978244 10.1038/sdata.2016.18 PMC4792175

[100] Culina A, Baglioni M, Crowther TW, Visser ME, Woutersen-Windhouwer S, Manghi P. Navigating the unfolding open data landscape in ecology and evolution. Nat Ecol Evol. 2018;2(3):420–6. 29453350 10.1038/s41559-017-0458-2

[101] Roche DG, Lanfear R, Binning SA, Haff TM, Schwanz LE, Cain KE, Kokko H, Jennions MD, Kruuk LE. Troubleshooting public data archiving: suggestions to increase participation. Plos Biol. 2014;12(1): e1001779. 24492920 10.1371/journal.pbio.1001779 PMC3904821

[102] Roche DG, Kruuk LEB, Lanfear R, Binning SA. Public data archiving in ecology and evolution: how well are we doing? Plos Biol. 2015;13(11): e1002295. 26556502 10.1371/journal.pbio.1002295 PMC4640582

[103] Culina A, van den Berg I, Evans S, Sanchez-Tojar A. Low availability of code in ecology: a call for urgent action. Plos Biol. 2020;18(7): e3000763. 32722681 10.1371/journal.pbio.3000763 PMC7386629

[104] Spake R, Mori AS, Beckmann M, Martin PA, Christie AP, Duguid MC, Doncaster CP. Implications of scale dependence for cross-study syntheses of biodiversity differences. Ecol Lett. 2021;24(2):374–90. 33216440 10.1111/ele.13641

[105] Osenberg CW, Sarnelle O, Cooper SD. Effect size in ecological experiments: the application of biological models in meta-analysis. Am Nat. 1997;150(6):798–812. 18811337 10.1086/286095

[106] Noble DWA, Nakagawa S. Planned missing data designs and methods: options for strengthening inference, increasing research efficiency and improving animal welfare in ecological and evolutionary research. Evol Appl. 2021;14(8):1958–68. 34429741 10.1111/eva.13273 PMC8372070

[107] Nakagawa S, Freckleton RP. Missing inaction: the dangers of ignoring missing data. Trends Ecol Evol. 2008;23(11):592–6. 18823677 10.1016/j.tree.2008.06.014

[108] Mavridis D, Chaimani A, Efthimiou O, Leucht S, Salanti G. Addressing missing outcome data in meta-analysis. Evid-Based Ment Health. 2014;17(3):85. 25009175 10.1136/eb-2014-101900 PMC13404265

[109] Ellington EH, Bastille-Rousseau G, Austin C, Landolt KN, Pond BA, Rees EE, Robar N, Murray DL. Using multiple imputation to estimate missing data in meta-regression. Methods Ecol Evol. 2015;6(2):153–63. 10.1111/2041-210X.12322

[110] Kambach S, Bruelheide H, Gerstner K, Gurevitch J, Beckmann M, Seppelt R. Consequences of multiple imputation of missing standard deviations and sample sizes in meta-analysis. Ecol Evol. 2020;10(20):11699–712. 33144994 10.1002/ece3.6806 PMC7593147

[111] Hadfield JD, Nakagawa S. General quantitative genetic methods for comparative biology: phylogenies, taxonomies and multi-trait models for continuous and categorical characters. J Evol Biol. 2010;23(3):494–508. 20070460 10.1111/j.1420-9101.2009.01915.x

[112] Cinar O, Nakagawa S, Viechtbauer W. Phylogenetic multilevel meta-analysis: a simulation study on the importance of modelling the phylogeny. Methods Ecol Evol. 2021. 10.1111/2041-210X.13760. 10.1111/2041-210X.13760

[113] Ives AR, Zhu J. Statistics for correlated data: phylogenies, space, and time. Ecol Appl. 2006;16(1):20–32. 16705958 10.1890/04-0702

[114] Maire A, Thierry E, Viechtbauer W, Daufresne M. Poleward shift in large-river fish communities detected with a novel meta-analysis framework. Freshwater Biol. 2019;64(6):1143–56. 10.1111/fwb.13291

[115] Morrissey MB. Meta-analysis of magnitudes, differences and variation in evolutionary parameters. J Evol Biol. 2016;29(10):1882–904. 27726237 10.1111/jeb.12950

[116] Cohen JM, Lajeunesse MJ, Rohr JR. A global synthesis of animal phenological responses to climate change. Nat Clim Change. 2018;8(3):224. 10.1038/s41558-018-0067-3

[117] Gurevitch J, Morrison JA, Hedges LV. The interaction between competition and predation: a meta-analysis of field experiments. Am Nat. 2000;155(4):435–53. 10753073 10.1086/303337

[118] Macartney EL, Lagisz M, Nakagawa S. The relative benefits of environmental enrichment on learning and memory are greater when stressed: a meta-analysis of interactions in rodents. Neurosci Biobehav R. 2022. 10.1016/j.neubiorev.2022.104554. 10.1016/j.neubiorev.2022.104554 35149103

[119] Spake R, Bowler DE, Callaghan CT, Blowes SA, Doncaster CP, Antão LH, Nakagawa S, McElreath R, Chase JM. Understanding ‘it depends’ in ecology: a guide to hypothesising, visualising and interpreting statistical interactions. Biol Rev. 2023. 10.1111/brv.12939. 10.1111/brv.12939 36859791

[120] Siviter H, Bailes EJ, Martin CD, Oliver TR, Koricheva J, Leadbeater E, Brown MJF. Agrochemicals interact synergistically to increase bee mortality. Nature. 2021;596(7872):389. 34349259 10.1038/s41586-021-03787-7

[121] Salanti G, Schmid CH. Research synthesis methods special issue on network meta-analysis: introduction from the editors. Res Synth Methods. 2012;3(2):69–70. 26062081 10.1002/jrsm.1050

[122] Gomez-Creutzberg C, Lagisz M, Nakagawa S, Brockerhoff EG, Tylianakis JM. Consistent trade-offs in ecosystem services between land covers with different production intensities. Biol Rev. 2021;96(5):1989–2008. 34031979 10.1111/brv.12734 PMC8519091

[123] Jackson D, White IR, Price M, Copas J, Riley RD. Borrowing of strength and study weights in multivariate and network meta-analysis. Stat Methods Med Res. 2017;26(6):2853–68. 26546254 10.1177/0962280215611702 PMC4964944

[124] Sanchez-Tojar A, Moran NP, O’Dea RE, Reinhold K, Nakagawa S. Illustrating the importance of meta-analysing variances alongside means in ecology and evolution. J Evol Biol. 2020;33(9):1216–23. 32512630 10.1111/jeb.13661

[125] Riley RD, Lambert PC, Abo-Zaid G. Meta-analysis of individual participant data: rationale, conduct, and reporting. BMJ. 2010;340:c221. 20139215 10.1136/bmj.c221

[126] Riley RD, Tierney JF, Stewart LA. Individual participant data meta-analysis : a handbook for healthcare research. 1st ed. Hoboken: Wiley; 2021.

[127] Hudson LN, Newbold T, Contu S, Hill SLL, Lysenko I, De Palma A, Phillips HRP, Alhusseini TI, Bedford FE, Bennett DJ, et al. The database of the PREDICTS (projecting responses of ecological diversity in changing terrestrial systems) project. Ecol Evol. 2017;7(1):145–88. 28070282 10.1002/ece3.2579 PMC5215197

[128] Dornelas M, Antao LH, Moyes F, Bates AE, Magurran AE, Adam D, Akhmetzhanova AA, Appeltans W, Arcos JM, Arnold H, et al. BioTIME: a database of biodiversity time series for the anthropocene. Glob Ecol Biogeogr. 2018;27(7):760–86. 30147447 10.1111/geb.12729 PMC6099392

[129] Mengersen K, Gurevitch J, Schmid CH. Meta-analysis of primary data. In: Koricheva J, Gurevitch J, Mengersen K, editors. Handbook of meta-analysis in ecology and evolution. Priceton: Princeton university; 2013. p. 300–12.

[130] Spake R, O’Dea RE, Nakagawa S, Doncaster CP, Ryo M, Callaghan CT, Bullock JM. Improving quantitative synthesis to achieve generality in ecology. Nat Ecol Evol. 2022;6(12):1818–28. 36329352 10.1038/s41559-022-01891-z

[131] Gerstner K, Moreno-Mateos D, Gurevitch J, Beckmann M, Kambach S, Jones HP, Seppelt R. Will your paper be used in a meta-analysis? Make the reach of your research broader and longer lasting. Methods Ecol Evol. 2017;8(6):777–84. 10.1111/2041-210X.12758

[132] Haddaway NR. A call for better reporting of conservation research data for use in meta-analyses. Conserv Biol. 2015;29(4):1242–5. 25588313 10.1111/cobi.12449

[133] Midolo G, De Frenne P, Holzel N, Wellstein C. Global patterns of intraspecific leaf trait responses to elevation. Global Change Biol. 2019;25(7):2485–98. 10.1111/gcb.14646 31056841

[134] White IR, Schmid CH, Stijnen T. Choice of effect measure and issues in extracting outcome data. In: Schmid CH, Stijnen T, White IR, editors. Handbook of meta-analysis. Boca Raton: CRC; 2021.

[135] Lajeunesse MJ. Bias and correction for the log response ratio in ecological meta-analysis. Ecology. 2015;96(8):2056–63. 26405731 10.1890/14-2402.1

