# Supplementary material for: Quantitative evidence synthesis: a practical guide on meta-analysis, meta-regression, and publication bias tests for environmental sciences
Source: Environ Evid. 2023 Apr 24;12:8. doi: 10.1186/s13750-023-00301-6 (PMC11378872; doi:10.1186/s13750-023-00301-6)
Supplement: Supplementary file 1 — Additional file 1: The survey of meta-analyses in environmnetal sciences. [file 13750_2023_301_MOESM1_ESM.html]

SURVEY – Environmental Evidence: State of meta-analytic practices


Code 

- Show All Code
- Hide All Code

# SURVEY – Environmental Evidence: State of meta-analytic practices

#### July 2022

## Aims

We aimed to locate and code 50 – 100 papers self-describing as containing meta-analysis, published in 2019 or 2020, classified as “Environmental Sciences” or “Environmental Studies”, in WoS database. These included papers should represent a diverse set of syntheses, from ecology and conservation to ecotoxicology and urban studies, but excluding studies focused on public health or occupational health, etc.

## Literature search

- Web of Science (Konwledge) ISI search engine on 12/01/2021.
- Search string covering years 2019 and 2020: (TS=(“meta\*analy\*” OR “meta\*regr\*” OR (quantitat\* W/5 \*review\* ) OR (quantitat\* W/5 \*synth\*) OR (global\* W/5 \*analy\*)) AND WC=(“environmental sciences” OR “environmental studies”) AND PY=(2019 OR 2020)) NOT WC=(” PUBLIC ENVIRONMENTAL OCCUPATIONAL HEALTH” ) Indexes: SCI-EXPANDED, SSCI, A&HCI, CPCI-S, CPCI-SSH, ESCI, CCR-EXPANDED, IC.
- Number of retrieved records: 434

## Literature search update

- The search was repeated using Web of Science (Konwledge) ISI search engine on 23/02/2022.
- Search string covering year 2021 only: (TS=(“meta\*analy\*” OR “meta\*regr\*” OR (quantitat\* W/5 \*review\* ) OR (quantitat\* W/5 \*synth\*) OR (global\* W/5 \*analy\*)) AND WC=(“environmental sciences” OR “environmental studies”) AND PY=(2021)) NOT WC=(” PUBLIC ENVIRONMENTAL OCCUPATIONAL HEALTH” ) Indexes: SCI-EXPANDED, SSCI, A&HCI, CPCI-S, CPCI-SSH, ESCI, CCR-EXPANDED, IC.
- Number of retrieved records: 293

## Inclusion criteria

1. Include studies claiming to be/contain meta-analysis (exclude protocols, methods, opinion articles, corrections, narrative reviews, etc.)
2. Include studies on the state of the natural or man/made environment (exclude exclusively human-focused studies)

## Abstract screening procedure and results

One researchers (ML) screened exported 434+293 bibliomteric records using Rayyan software, resulting in 56+ provisionaly included articles (available in files *included\_articles\_rayyan\_pilot3.ris* and *included\_articles\_rayyan\_pilot3.csv*; for search update: ). Further 3 articles were initially excluded from the 2019-2020 search, as their actual publication date was in 2021 (captured as Online Early in 2020 records), but were captured by the updated search in 2022.

## Full text screening and data extraction

Provisionaly included full-text articles were further screened and extracted pre-determined data using piloted online form implemented as a GoogleForm. This form consisted of two parts: 1) paper info and screening of full text using the inclusion criteria, and 2) extracting data on meta-analytic methods and transparency of reporting (see meta-data). Randomly selected 3 papers were initially screened and extracted independently by to researchers (ML and EM) to pilot the form. Then, the remaining papers were split evenly between the two researchers. All data extractions were be cross-checked by the other researcher and any potential disagreements were resolved by discussion, involving an independent reviewer (SN) where needed. Articles from the search update in 2022, were extracted by ML. Then all articles were cross-checked by YY, involving a fourth reviewer (SN) where needed. Final data extraction spreadsheet was exported and saved as *MA\_EnvironResearch\_Survey\_3 (Responses).csv* .

Results presented below are based on data extraction from 82 papers.

```
rawdata <- read.csv(here("data", "MA_EnvironResearch_Survey_3 (Responses).csv"), check.names = FALSE)
#dim(rawdata) #82 papers
#names(rawdata)
#glimpse(rawdata) # taking a quick look
```

```
## Add a column coding which studies were included or excluded
rawdata %>% 
  mutate(
    Included = case_when(
      str_detect(rawdata[,7], "no") ~ "no", 
      str_detect(rawdata[,9], "no") ~ "no", 
      str_detect(rawdata[,11], "no") ~ "no", 
      TRUE ~ "yes"
    )
  ) -> rawdata
#table(rawdata$Included) # no = 9, yes = 73

## Add a column coding main exclusion reasons
rawdata %>% 
  mutate(
    Exclusion_reason = case_when(
      str_detect(rawdata[,7], "no") ~ "Not in English", 
      str_detect(rawdata[,9], "no") ~ "Not self-describing as a meta-analysis", 
      str_detect(rawdata[,11], "no") ~ "Not focused on environmental topics", 
      TRUE ~ "NA"
    )
  ) -> rawdata

#table(rawdata$Exclusion_reason)
```

### Summary tables

There are 73 included papers and 9 excluded papers.

#### Table S1

List of excluded articles with exclusion reasons.

```
## Remove all included studies and select a few relevant columns
rawdata_excl <- rawdata %>% filter(Included == "no") %>% select(c(4, 5, 6, 3, 13,47)) 
#names(rawdata_excl)
names(rawdata_excl) <- c("First_author", "Year", "Journal", "Title", "DOI", "Exclusion_reason")

## Make a summary table of excluded studies
t1 <- rawdata_excl %>%   DT::datatable(rownames = FALSE, width = "100%", options = list(dom = 't', scrollY = '700px', pageLength = 20))
t1
```

#### Table S2

List of included articles.

```
## Remove rows with excluded studies and remove first one and last two columns, then all columns with "Comment","checked"
rawdata_incl <- rawdata %>% filter(Included == "yes") %>% select(-c(1, 46:47)) %>% select(-starts_with("Comment")) %>% select(-starts_with("checked")) 

## Make a summary table of included studies
rawdata_incl2 <- select(rawdata_incl, c(3,4,5,2,9)) 
names(rawdata_incl2) <- c("First_author", "Year", "Journal", "Title", "DOI")
t2 <- rawdata_incl2 %>% DT::datatable(rownames = FALSE, width = "100%", options = list(dom = 't', scrollY = '700px', pageLength = 80))
t2
```

#### Table S3

List of data extraction questions and answer options.

```
## Create a table of data extraction questions for included studies

## Create a column of questions - based on column names in teh main data frame
Questions <- str_sort(names(rawdata_incl)) #save old names as these are our data extraction questions and sort by number

## Create a new column showing what type of answers can be recorded for each questions
Options <- c("free text", 
             "free text", 
             "free text", 
             "number", 
             "free text", 
             "yes; no", 
             "yes; no; unclear/other", 
             "yes; no; unclear/other", 
             "DOI", 
             "SMD (d, g, etc.); correlation (r); Response Ratio (lnRR); Odds Ratio (OR); p-value; coefficient of variation ratio (lnCVR); variance ratio (VR); proportion/percentage; mean (unstandardised); mean difference (unstandardised); unclear/other", 
             "yes; no; unclear/other", 
             "fixed-effect (meta-analytic) model; random-effects (meta-analytic) model; multilevel (hierarchical) (meta-analytic) model; unweighted regression model; weighted regression model; unweighted mixed-effects (hierarchical) model; weighted mixed-effects (hierarchical) model; vote counting; unclear/other; N/A (no models)",
             "weighted model; unweighted model; unclear/other model", 
             "paper explicitly models non-independence (at least some of it); paper deals with non-independence in other than modeling way (e.g., aggregation, sampling); paper only mentions non-independence (e.g., in limitations); unclear/other/not mentioned; N/A (they claim all data are independent)", 
             "yes; no; unclear/other", 
             "yes; no; not applicable", 
             "no; yes, it uses graphical tests (e.g., funnel plot); yes, it uses regression test (e.g. Egger's regression); yes, it uses trim-and-fill test; yes, it uses failsafe number; unclear/other",
             "yes; no; unclear/other; N/A (e.g. they conducted unweighted meta-analysis)", 
             "yes; no; unclear/other", 
             "VCV; RVE; no; unclear/other; N/A (independent as they made dataset independent or they did not model e.g. vote-counting)", 
             "yes; no; unclear/other", 
             "yes; no; unclear/other", 
             "R metafor; R MCMCglmm; R meta; R lm (basic); R lmer (lme4) or lme (nlme); MetaWin; OpenMEE; unclear/other", 
             "yes; no", 
             "yes; no") 

t3 <- tibble(Questions, Options) %>% DT::datatable(rownames = FALSE, width = "100%", options = list(dom = 't', scrollY = '700px', pageLength = 25))
t3
```

### Data cleaning for plotting

More data cleaning before plotting.

```
## Replace column names with shorter variable names for rawdata_incl analyses
names(rawdata_incl)  <- c("Extractor",
                 "Title",
                 "Author",
                 "Year",
                 "Journal",
                 "English",
                 "Meta_analysis",
                 "Environmental",
                 "DOI",
                 "Effect_size",
                 "Multiple_ES_per_study",
                 "Statistical_models",
                 "Weighted_models",
                 "Non_independence_modelled",
                 "Outcomes_per_area",
                 "Area_accounted_for",
                 "Publication_bias_tested",
                 "Heterogenity_reported",
                 "Sensitivity_tested",
                 "Error_non_independence",
                 "Within_study_bias",
                 "Included_studies_listed",
                 "Software_used",
                 "Code_provided",
                 "Data_provided")

#names(rawdata_incl)
```

### Plots

#### Publication year

```
#table(rawdata_incl$Year)

count(rawdata_incl, Year) %>%
  arrange(n) %>%
  mutate(class = factor(Year, levels = Year)) %>%
  ggplot(aes(x = class, y = n)) +
  geom_bar(stat = "identity", position = "dodge") +
  geom_text(aes(label=scales::comma(n)), hjust = 0, nudge_y = 1) +
  coord_flip() +
  scale_y_continuous(breaks = seq(0,80,5)) +
  labs(x = "", y = "Article count", title = "When it was published?")
```

```
# ggsave(filename = "./figs/FigS1.pdf",
#        plot = plot_qual,
#        height = 5, width = 10,
#        device = cairo_pdf)
```

#### Publication journal

```
#table(rawdata_incl$Journal)
rawdata_incl$Journal <- gsub("Naturee Sustainability", "Nature Sustainability", rawdata_incl$Journal) #fix typo

count(rawdata_incl, Journal) %>%
  arrange(n) %>%
  mutate(class = factor(Journal, levels = Journal)) %>%
  ggplot(aes(x = class, y = n)) +
  geom_bar(stat = "identity", position = "dodge") +
  geom_text(aes(label = scales::comma(n)), hjust = 0, nudge_y = 1) +
  coord_flip() +
  scale_y_continuous(breaks=seq(0,80,5)) +
  labs(x = "", y = "Article count", title = "Where it was published?")
```

```
# ggsave(filename = "./figs/FigS2.pdf",
#        plot = plot_qual,
#        height = 12, width = 10,
#        device = cairo_pdf)
```

#### Effect size type

```
#table(rawdata_incl$Effect_size) 
rawdata_incl$Effect_size <- as.factor(rawdata_incl$Effect_size)

##Simplify effect size names
levels(rawdata_incl$Effect_size) <- c("correlation", 
                                      "mean", 
                                      "OR", 
                                      "proportion/percentage", 
                                      "proportion/percentage and mean", 
                                      "lnRR", 
                                      "lnRR and proportion/percentage", 
                                      "lnRR and unclear/other", 
                                      "SMD", 
                                      "SMD and lnRR", 
                                      "SMD and unclear/other",
                                      "unclear/other")

Effect_size <- separate_rows(rawdata_incl, Effect_size, sep = " and ") #split rows with multiple values

count(Effect_size, Effect_size) %>%
  arrange(n) %>%
  mutate(class = factor(Effect_size, levels = Effect_size)) %>%
  ggplot(aes(x = class, y = n)) +
  geom_bar(stat = "identity", position = "dodge") +
  geom_text(aes(label = scales::comma(n)), hjust = 0, nudge_y = 1) +
  coord_flip() +
  scale_y_continuous(breaks=seq(0,80,5)) +
  labs(x = "", y = "Article count", title = "What type of effect size was used?", caption = "Note: some studies used more than one")
```

```
# ggsave(filename = "./figs/FigS3.pdf",
#        plot = plot_qual,
#        height = 5, width = 10,
#        device = cairo_pdf)
```

#### Multiple ES per study

Indicating potential data non-independence in the models when k > N (number of effect sizes larger than number of included articles).

```
#table(rawdata_incl$Multiple_ES_per_study)
rawdata_incl$Multiple_ES_per_study <- as.factor(rawdata_incl$Multiple_ES_per_study)
levels(rawdata_incl$Multiple_ES_per_study) <- c("unclear/other", "yes")

count(rawdata_incl, Multiple_ES_per_study) %>%
  arrange(n) %>%
# mutate(class = factor("no")) %>% #used when only "no" was coded for all studies
  mutate(class = factor(Multiple_ES_per_study, levels = Multiple_ES_per_study)) %>%
  ggplot(aes(x = class, y = n)) +
  geom_bar(stat = "identity", position = "dodge") +
  geom_text(aes(label = scales::comma(n)), hjust = 0, nudge_y = 1) +
  coord_flip() +
  scale_y_continuous(breaks = seq(0,80,5)) +
  labs(x = "", y = "Article count", title = "Multiple ES per study?") #, caption = "Literature survey result"
```

```
# ggsave(filename = "./figs/FigS4.pdf",
#        plot = plot_qual,
#        height = 3, width = 10,
#        device = cairo_pdf)
```

#### Statistical models

```
#table(rawdata_incl$Statistical_models)
rawdata_incl$Statistical_models <- as.factor(rawdata_incl$Statistical_models)
levels(rawdata_incl$Statistical_models) <- c("fixed-effect (meta-analytic) model",
                                             "fixed-effect (meta-analytic) model and random-effects (meta-analytic) model",
                                             "multilevel (hierarchical) (meta-analytic) model",                         
                                             "N/A (no models)",                                                       
                                             "random-effects (meta-analytic) model",                                    
                                             "random-effects (meta-analytic) model and unweighted regression model",       
                                             "random-effects (meta-analytic) model and vote counting",                     
                                             "unweighted mixed-effects (hierarchical) model",                           
                                             "unweighted regression model",                                             
                                             "weighted mixed-effects (hierarchical) model",                             
                                             "weighted regression model") 

Statistical_models <- separate_rows(rawdata_incl, Statistical_models, sep = " and ") #split rows with multiple values

count(Statistical_models, Statistical_models) %>%
  arrange(n) %>%
  mutate(class = factor(Statistical_models, levels = Statistical_models)) %>%
  ggplot(aes(x = class, y = n)) +
  geom_bar(stat = "identity", position = "dodge") +
  geom_text(aes(label = as.integer(scales::comma(n))), hjust = 0, nudge_y = 1) +
  coord_flip() +
  scale_y_continuous(breaks = seq(0,80,5)) +
  labs(x = "", y = "Article count", title = "What type of statistical model was used?", caption = "Note: some studies used more than one")
```

```
# ggsave(filename = "./figs/FigS5.pdf",
#        plot = plot_qual,
#        height = 8, width = 10,
#        device = cairo_pdf)
```

#### Weighted

```
#table(rawdata_incl$Weighted)
rawdata_incl$Weighted <- as.factor(rawdata_incl$Weighted)
levels(rawdata_incl$Weighted) <- c("unclear/other", "unweighted model", "weighted model")

count(rawdata_incl, Weighted) %>%
  arrange(n) %>%
  mutate(class = factor(Weighted, levels = Weighted)) %>%
  ggplot(aes(x = class, y = n)) +
  geom_bar(stat = "identity", position = "dodge") +
  geom_text(aes(label=as.integer(scales::comma(n))), hjust = 0, nudge_y = 1) +
  coord_flip() +
  scale_y_continuous(breaks = seq(0,80,5)) +
  labs(x = "", y = "Article count", title = "Did it use a weighted model?") #, caption="Literature survey result"
```

```
# ggsave(filename = "./figs/FigS6.pdf",
#        plot = plot_qual,
#        height = 5, width = 10,
#        device = cairo_pdf)
```

#### Non-independence modelled

```
#table(rawdata_incl$Non_independence_modelled)
rawdata_incl$Non_independence_modelled <- as.factor(rawdata_incl$Non_independence_modelled)
levels(rawdata_incl$Non_independence_modelled) <- c("N/A (claimed independence)",
                                                    "non-modelling way",
                                                    "explicit modelling",
                                                    "only mentioned", 
                                                    "unclear/other")

Non_independence_modelled_df <- separate_rows(rawdata_incl, Non_independence_modelled, sep = " and ") #split rows with multiple values

count(Non_independence_modelled_df, Non_independence_modelled) %>%
  arrange(n) %>%
  mutate(class = factor(Non_independence_modelled, levels = Non_independence_modelled)) %>%
  ggplot(aes(x = class, y = n)) +
  geom_bar(stat = "identity", position = "dodge") +
  geom_text(aes(label = as.integer(scales::comma(n))), hjust = 0, nudge_y = 1) +
  coord_flip() +
  scale_y_continuous(breaks = seq(0,80,5)) +
  labs(x = "", y = "Article count", title = " Did it model non-independence?") #, caption="Literature survey result"
```

```
# ggsave(filename = "./figs/FigS7.pdf",
#        plot = plot_qual,
#        height = 6, width = 10,
#        device = cairo_pdf)
```

#### Outcomes per area

```
#table(rawdata_incl$Outcomes_per_area)
rawdata_incl$Outcomes_per_area <- recode(rawdata_incl$Outcomes_per_area, "unclear/other/not mentioned" = "unclear/other")

count(rawdata_incl, Outcomes_per_area) %>%
  arrange(n) %>%
  mutate(class = factor(Outcomes_per_area, levels = Outcomes_per_area)) %>%
  ggplot(aes(x = class, y = n)) +
  geom_bar(stat = "identity", position = "dodge") +
  geom_text(aes(label = scales::comma(n)), hjust = 0, nudge_y = 1) +
  coord_flip() +
  scale_y_continuous(breaks = seq(0,80,5)) +
  labs(x = "", y = "Article count", title = "Were outcomes measured over an area?") #, caption="Literature survey result"
```

```
# ggsave(filename = "./figs/FigS8.pdf",
#        plot = plot_qual,
#        height = 5, width = 10,
#        device = cairo_pdf)
```

#### Area accounted for

```
#table(rawdata_incl$Area_accounted_for)
rawdata_incl$Area_accounted_for <- as.factor(rawdata_incl$Area_accounted_for)
levels(rawdata_incl$Area_accounted_for) <- c("no","N/A (not per area)", "yes")

count(rawdata_incl, Area_accounted_for) %>%
  arrange(n) %>%
  mutate(class = factor(Area_accounted_for, levels = Area_accounted_for)) %>%
  ggplot(aes(x = class, y = n)) +
  geom_bar(stat = "identity", position = "dodge") +
  geom_text(aes(label=as.integer(scales::comma(n))), hjust = 0, nudge_y = 1) +
  coord_flip() +
  scale_y_continuous(breaks = seq(0,80,5)) +
  labs(x = "", y = "Article count", title = "Was the sampling area accounted for?") #, caption="Literature survey result"
```

```
# ggsave(filename = "./figs/FigS9.pdf",
#        plot = plot_qual,
#        height = 5, width = 10,
#        device = cairo_pdf)
```

#### Publication bias

```
#table(rawdata_incl$Publication_bias_tested)
rawdata_incl$Publication_bias_tested <- as.factor(rawdata_incl$Publication_bias_tested)
levels(rawdata_incl$Publication_bias_tested) <- c("not tested",
                                                  "unclear/other",
                                                  "failsafe number",
                                                  "graphical test",
                                                  "graphical test and unclear/other",
                                                  "graphical test and failsafe number",
                                                  "graphical test and failsafe number and unclear/other",
                                                  "graphical test and regression test",
                                                  "graphical test and regression test and failsafe number",
                                                  "graphical test and regression test and trim-and-fill test",
                                                  "graphical test and regression test and trim-and-fill test and failsafe number",
                                                  "graphical test and trim-and-fill test",
                                                  "graphical test and trim-and-fill test and failsafe number",
                                                  "regression test",
                                                  "regression test and trim-and-fill test",
                                                  "trim-and-fill test and failsafe number")

Publication_bias_tested <- separate_rows(rawdata_incl, Publication_bias_tested, sep = " and ") #split rows with multiple values

count(Publication_bias_tested, Publication_bias_tested) %>%
  arrange(n) %>%
  mutate(class = factor(Publication_bias_tested, levels = Publication_bias_tested)) %>%
  ggplot(aes(x = class, y = n)) +
  geom_bar(stat = "identity", position = "dodge") +
  geom_text(aes(label = as.integer(scales::comma(n))), hjust = 0, nudge_y = 1) +
  coord_flip() +
  scale_y_continuous(breaks=seq(0,40,5)) +
  labs(x = "", y = "Article count", title = "What type of publication bias was used?", caption = "Note: some studies used more than one")
```

```
# ggsave(filename = "./figs/FigS10.pdf",
#        plot = plot_qual,
#        height = 8, width = 10,
#        device = cairo_pdf)
```

#### Heterogeneity

```
#table(rawdata_incl$Heterogenity_reported)
rawdata_incl$Heterogenity_reported <- as.factor(rawdata_incl$Heterogenity_reported)
levels(rawdata_incl$Heterogenity_reported) <- c("N/A (not weighted models)", "no", "yes")

count(rawdata_incl, Heterogenity_reported) %>%
  arrange(n) %>%
  mutate(class = factor(Heterogenity_reported, levels = Heterogenity_reported)) %>%
  ggplot(aes(x = class, y = n)) +
  geom_bar(stat = "identity", position = "dodge") +
  geom_text(aes(label = as.integer(scales::comma(n))), hjust = 0, nudge_y = 1) +
  coord_flip() +
  scale_y_continuous(breaks = seq(0,80,5)) +
  labs(x = "", y = "Article count", title = "Was heterogeneity measure reported?")#, caption="Note: N/A - not applicable"
```

```
# ggsave(filename = "./figs/FigS11.pdf",
#        plot = plot_qual,
#        height = 5, width = 10,
#        device = cairo_pdf)
```

#### Sensitivity analyses

```
#table(rawdata_incl$Sensitivity_tested)

count(rawdata_incl, Sensitivity_tested) %>%
  arrange(n) %>%
  mutate(class = factor(Sensitivity_tested, levels = Sensitivity_tested)) %>%
  ggplot(aes(x = class, y = n)) +
  geom_bar(stat = "identity", position = "dodge") +
  geom_text(aes(label = as.integer(scales::comma(n))), hjust = 0, nudge_y = 1) +
  coord_flip() +
  scale_y_continuous(breaks = seq(0,80,5)) +
  labs(x = "", y = "Article count", title = "Were sensitivity analyses performed?") #, caption="Literature survey result"
```

```
# ggsave(filename = "./figs/FigS12.pdf",
#        plot = plot_qual,
#        height = 5, width = 10,
#        device = cairo_pdf)
```

#### Non-independence among sampling errors modelled

```
#table(rawdata_incl$Error_non_independence)
rawdata_incl$Error_non_independence <- as.factor(rawdata_incl$Error_non_independence)
levels(rawdata_incl$Error_non_independence) <- c("N/A (independent or no models)",
                                                    "no",
                                                    "RVE",
                                                    "unclear/other", 
                                                    "VCV")

Error_non_independence_df <- separate_rows(rawdata_incl, Error_non_independence, sep = " and ") #split rows with multiple values

count(Error_non_independence_df, Error_non_independence) %>%
  arrange(n) %>%
  mutate(class = factor(Error_non_independence, levels = Error_non_independence)) %>%
  ggplot(aes(x = class, y = n)) +
  geom_bar(stat = "identity", position = "dodge") +
  geom_text(aes(label = as.integer(scales::comma(n))), hjust = 0, nudge_y = 1) +
  coord_flip() +
  scale_y_continuous(breaks = seq(0,80,5)) +
  labs(x = "", y = "Article count", title = " Did it model error non-independence?") #, caption="Literature survey result"
```

```
# ggsave(filename = "./figs/FigS13.pdf",
#        plot = plot_qual,
#        height = 6, width = 10,
#        device = cairo_pdf)
```

#### (Within-study) risk of bias assessed

```
#table(rawdata_incl$Within_study_bias)

count(rawdata_incl, Within_study_bias) %>%
  arrange(n) %>%
  mutate(class = factor(Within_study_bias, levels = Within_study_bias)) %>%
  ggplot(aes(x = class, y = n)) +
  geom_bar(stat = "identity", position = "dodge") +
  geom_text(aes(label = as.integer(scales::comma(n))), hjust = 0, nudge_y = 1) +
  coord_flip() +
  scale_y_continuous(breaks = seq(0,80,5)) +
  labs(x = "", y = "Article count", title = " Did it assess within-study risk of bias?") #, caption="Literature survey result"
```

```
# ggsave(filename = "./figs/FigS14.pdf",
#        plot = plot_qual,
#        height = 3, width = 10,
#        device = cairo_pdf)
```

#### List of included studies provided

```
#table(rawdata_incl$Included_studies_listed)
rawdata_incl$Included_studies_listed <- as.factor(rawdata_incl$Included_studies_listed)
levels(rawdata_incl$Included_studies_listed) <- c("no", "unclear/other", "yes")

count(rawdata_incl, Included_studies_listed) %>%
  arrange(n) %>%
  mutate(class = factor(Included_studies_listed, levels = Included_studies_listed)) %>%
  ggplot(aes(x = class, y = n)) +
  geom_bar(stat = "identity", position = "dodge") +
  geom_text(aes(label = as.integer(scales::comma(n))), hjust = 0, nudge_y = 1) +
  coord_flip() +
  scale_y_continuous(breaks=seq(0,80,5)) +
  labs(x = "", y = "Article count", title = "Is the list of included articles provided?") #, caption="Literature survey result"
```

```
# ggsave(filename = "./figs/FigS15.pdf",
#        plot = plot_qual,
#        height = 5, width = 10,
#        device = cairo_pdf)
```

#### Software used

```
#table(rawdata_incl$Software_used) 
rawdata_incl$Software_used <- as.factor(rawdata_incl$Software_used)
levels(rawdata_incl$Software_used) <- c("MetaWin",
                                        "OpenMEE",
                                        "R lme4 or nlme",
                                        "R metafor",
                                        "unclear/other")

count(rawdata_incl, Software_used) %>%
  arrange(n) %>%
  mutate(class = factor(Software_used, levels = Software_used)) %>%
  ggplot(aes(x = class, y = n)) +
  geom_bar(stat = "identity", position = "dodge") +
  geom_text(aes(label = as.integer(scales::comma(n))), hjust = 0, nudge_y = 1) +
  coord_flip() +
  scale_y_continuous(breaks = seq(0,80,5)) +
  labs(x = "", y = "Article count", title = "What type of statistical software was used?", caption = "Note: some studies used more than one")
```

```
# ggsave(filename = "./figs/FigS16.pdf",
#        plot = plot_qual,
#        height = 8, width = 10,
#        device = cairo_pdf)
```

#### Code availability

```
#table(rawdata_incl$Code_provided)

count(rawdata_incl, Code_provided) %>%
  arrange(n) %>%
  mutate(class = factor(Code_provided, levels = Code_provided)) %>%
  ggplot(aes(x = class, y = n)) +
  geom_bar(stat = "identity", position = "dodge") +
  geom_text(aes(label = scales::comma(n)), hjust = 0, nudge_y = 1) +
  coord_flip() +
  scale_y_continuous(breaks = seq(0,80,5)) +
  labs(x = "", y = "Article count", title = "Is analysis code provided?") #, caption="Literature survey result"
```

```
# ggsave(filename = "./figs/FigS17.pdf",
#        plot = plot_qual,
#        height = 3, width = 10,
#        device = cairo_pdf)
```

#### Data availability

```
#table(rawdata_incl$Data_provided)

count(rawdata_incl, Data_provided) %>%
  arrange(n) %>%
  mutate(class = factor(Data_provided, levels = Data_provided)) %>%
  ggplot(aes(x = class, y = n)) +
  geom_bar(stat = "identity", position = "dodge") +
  geom_text(aes(label = scales::comma(n)), hjust = 0, nudge_y = 1) +
  coord_flip() +
  scale_y_continuous(breaks = seq(0,80,5)) +
  labs(x = "", y = "Article count", title = "Is data provided?") #, caption="Literature survey result"
```

```
# ggsave(filename = "./figs/FigS18.pdf",
#        plot = plot_qual,
#        height = 3, width = 10,
#        device = cairo_pdf)
```
